# The ability of single genes vs full genomes to resolve time and space in outbreak analysis

**DOI:** 10.1186/s12862-019-1567-0

**Published:** 2019-12-26

**Authors:** Gytis Dudas, Trevor Bedford

**Affiliations:** 1 Fred Hutchinson Cancer Research Center, 1100 Fairview Ave N, Seattle, 98109 USA; 2 Gothenburg Global Biodiversity Centre, Carl Skottsbergs gata 22B, Gothenburg, 413 19 Sweden

**Keywords:** Phylogenetics, Phylogeography, Genomic epidemiology, Ebola virus

## Abstract

**Background:**

Inexpensive pathogen genome sequencing has had a transformative effect on the field of phylodynamics, where ever increasing volumes of data have promised real-time insight into outbreaks of infectious disease. As well as the sheer volume of pathogen isolates being sequenced, the sequencing of whole pathogen genomes, rather than select loci, has allowed phylogenetic analyses to be carried out at finer time scales, often approaching serial intervals for infections caused by rapidly evolving RNA viruses. Despite its utility, whole genome sequencing of pathogens has not been adopted universally and targeted sequencing of loci is common in some pathogen-specific fields.

**Results:**

In this study we highlighted the utility of sequencing whole genomes of pathogens by re-analysing a well-characterised collection of Ebola virus sequences in the form of complete viral genomes (≈19 kb long) or the rapidly evolving glycoprotein (GP, ≈2 kb long) gene. We have quantified changes in phylogenetic, temporal, and spatial inference resolution as a result of this reduction in data and compared these to theoretical expectations.

**Conclusions:**

We propose a simple intuitive metric for quantifying temporal resolution, i.e. the time scale over which sequence data might be informative of various processes as a quick back-of-the-envelope calculation of statistical power available to molecular clock analyses.

## Background

The combination of decreasing cost of sequencing and the unparalleled insight it offers have led to the adoption of pathogen genetic sequencing as one of the most effective tools in a modern epidemiologist’s toolkit. When coupled with sophisticated models of evolution pathogen sequences can be used to look into epidemiological features such as cryptic transmission [1], migration [2, 3], and origins [4] of infectious diseases amongst others. Pathogen sequences also contain information about past temporal dynamics before sequence data have been collected [5] due to the pattern of shared and unique mutations inherited from preceding generations. Molecular phylogenetic approaches rely on decoding these patterns of shared mutations into a nested graph known as the phylogenetic tree. Pathogens often have short generation times and some, like RNA viruses, also possess polymerases with low replication fidelity such that mutations are generated at a rapid pace [6, 7] leading to fast differentiation of pathogen lineages at the genetic level as they spread. With appropriate sampling and information (“metadata”) about sequences historic population dynamics can be inferred and quantified from pathogen phylogenies. Changes in pathogen population sizes over time [8], inference of unobserved ancestral states [9], correlates of processes [10, 11], and overall phylodynamic [12] patterns can be inferred from molecular phylogenies and used to understand patterns of pathogen transmission at a number of scales.

Before widespread adoption of high-throughput sequencing limitations and costs led to amplification and sequencing of short fragments of pathogen genomes [13, 14]. These subgenomic fragments were often chosen for their diversity such as viral surface glycoproteins that experience selective pressures from vertebrate immune systems or their utility such as routine sequencing of Human immunodeficiency virus (HIV) *pol* gene to test for drug resistance [15, 16]. Whilst subgenomic fragments of pathogens are very accurate and specific as diagnostic markers and informative about long-term evolution their length (dictated by the compromise between information content and ease of sequencing) limits their utility in detailed molecular epidemiology investigations for example during outbreaks [17] as only mutations occurring within the small region of the genome that is sequenced are available for phylogenetic inference.

Molecular clocks have been particularly useful in molecular epidemiology where the accumulation of mutations between sequences is used as a noisy approximation for elapsed time, given either times of events in the phylogeny (sequence dates or dates of common ancestors) or a previously determined molecular clock rate. Generally neutral pathogen variation at the nucleotide level ebbs and flows under the forces of population genetics unlike beneficial or deleterious variation which tends to either fix or be purged rapidly, respectively. Due to their random and discrete nature mutations are modelled as a Poisson process [18] where the waiting time *t* for observing a mutation at a single site is exponentially distributed with evolutionary rate parameter *R*. The probability of observing 0 mutations at a single site after time *t* is *e*^−*Rt*^ and the probability of at least one mutation is therefore 1−*e*^−*Rt*^. Higher evolutionary rates *R* or waiting times *t* result in higher probabilities of observing at least one mutation at the site in question. Since sites are assumed to evolve independently the probability of observing at least one mutation across *L* sites is

1 $$ P = 1-e^{-RLt},  $$

where *RL* is expressed in substitutions per year (rate in substitutions per site per year multiplied by number of sites). Since the probability of observing at least one mutation changes depending on waiting time (and *vice versa*) we instead focus on the mean waiting time until at least one mutation appears. The mean of an exponential distribution is *λ*^−1^ where *λ* in our case is *RL* such that mean waiting time $\bar {t}$ under a given evolutionary rate *R* and sequence length *L* becomes

2 $$ \bar{t} = \frac{1}{RL}   $$

When the evolutionary rate *R* or sequence length *L* are low mean waiting times $\bar {t}$ are lengthened and *vice versa*. It also suggests a worrying relationship between *R*, *L*, and $\bar {t}$ - a reduction in either *R* or *L* leads to a reciprocal reduction in $\bar {t}$ such that reducing the number of sites by 10%, for example, requires a $\frac {1}{0.9} = 1.11(1)$-fold increase in evolutionary rate to maintain the same temporal resolution $\bar {t}$ or risk increasing the waiting time for a mutation by the same amount (i.e. ≈11% or 0.9 of the original resolution available). This gets worse reciprocally such that halving the sites requires doubling the evolutionary rate, using 10% of the sites requires a ten-fold increase in evolutionary rate, *etc*.

Since both maximum plausible evolutionary rates *R* and genome length *G* are largely dictated by deleterious mutation load neither quantity will vary substantially for a given pathogen though individually *R* and *G* can vary substantially where for example viruses have high *R* and low *G* on average and bacteria have higher *G* but lower *R*. Sequencing recovers some fraction *f* of the genome length *G* (*L*=*Gf*) for analyses and sequencing complete genomes (*f*=1.0;*L*=*G*) is the best possible scenario since sequencing any shorter region requires the evolutionary rate to increase by a factor of $\frac {1}{f}$ which even if *L*=0.4*G* means the evolutionary rate would have to be 2.5 times faster in the remaining 0.4 of the genome to be able to record information in the form of mutations at the same speed as complete genomes. The message of our manuscript, at least as far as densely sampled infectious disease outbreaks go, is that the task of sequencing a complete pathogen genome will rarely be as miserable a task as analysing a fraction of one.

In this study we show this by quantifying how much information relevant to phylodynamic analysis is lost when shorter genomic regions are used instead of full genomes. By focusing our attention on a subset (600 sequences) of a well-characterised genomic sequence data (comprised of >1600 viral genomes) set derived from the West African Ebola virus epidemic of 2013-2015 [11] we estimate loss in precision and accuracy of molecular clock models and phylogeographic inference methods when only the glycoprotein gene (GP), a region representing just 10% of the viral genome, is analysed despite GP evolving at rates faster than the genomic average. Our methods rely on masking tip dates and locations for 60 (10%) of the sequences in a classic training-testing split where we re-infer these parameters as latent variables using Markov chain Monte Carlo (MCMC). We show that this reduction in data not only leads to severe mixing issues in MCMC analyses by removing the constraints additional data impose on plausible parameter space without adding restrictive priors to compensate, but can also result in unreliable tip date and location inference. Despite achieving much better temporal resolution when using complete viral genomes we still find residual error caused by inherent randomness of mutations which is close to theoretical expectations (Eq 2). We refer to this as the temporal horizon, i.e. a temporal resolution limit where population processes occurring at a rate faster than the rate at which mutations enter and are observed in a population will not be captured with high fidelity even with genome sequences.

## Results

### Loss of phylogenetic signal

**Fig. 1:**
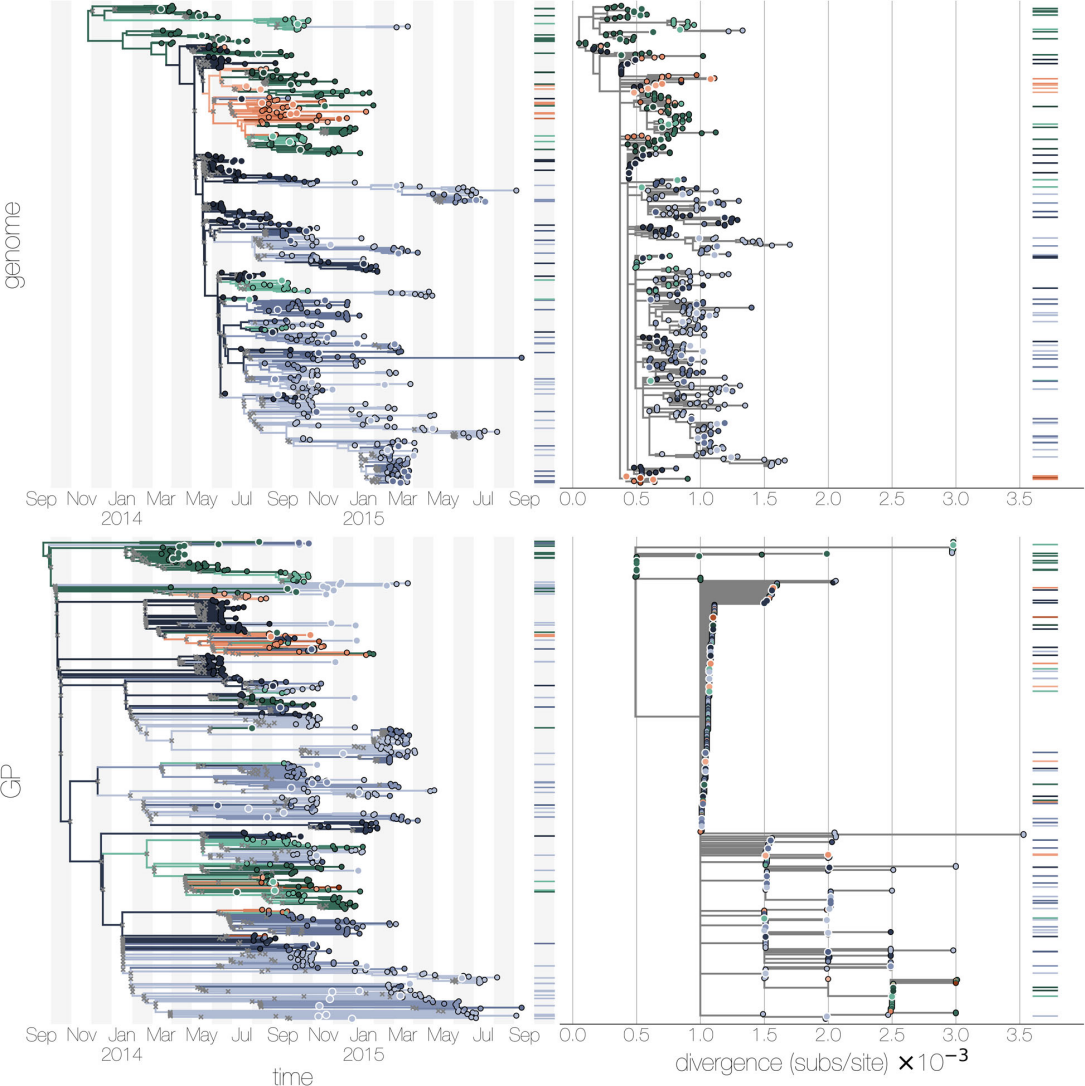
Phylogenies of West African Ebola virus genomes (top) or GP sequences (bottom). Temporal phylogenies recovered using BEAST are shown on the left and maximum likelihood phylogenies recovered with RAxML are on the right. Tips are coloured based on country (Sierra Leone in blue, Liberia in red, Guinea in green) and location (lighter colours indicate administrative divisions lying towards west of the country). Tips outlined in white indicate the 60 chosen for date and location masking, ticks to the right of phylogenies indicate the y positions of masked tips coloured by their true location. In temporal phylogenies branches are also coloured based on GLM-inferred ancestral locations. Nodes in temporal phylogenies with <0.10 posterior probability are indicated with grey X marks. Maximum likelihood phylogenies on the right are rooted via temporal regression in TreeTime

Figure 1 shows the reconstructed phylogenies in substitution space (right) and time space (left) for 600 complete Ebola virus genomes (top) or just GP sequences (bottom). Although higher levels of divergence are observed in the GP dataset, as seen from tree height, the differences in the number of non-polytomic nodes between genomic and GP data are clear, indicating substantially better resolution in disentangling the exact relationships between lineages in the former. Additional file 1 shows where in the better resolved maximum likelihood phylogeny of genome sequences the mutations that occurred in just the region spanning the GP gene are located with continuous blocks of colours corresponding to regions of the tree that would become collapsed if only the GP data were used and the tree were inferred correctly. Internal branches of a phylogeny correspond to hypotheses of common ancestry and in the case of GP only 42 internal nodes are identified in the maximum likelihood phylogeny compared to 210 internal nodes for complete genomes. The one aspect of the West African epidemic that can be inferred from both GP and genome phylogenies is that the virus’ origins lie in Guinea but details of its onwards spread are largely lost in the GP phylogeny. Genomic data, on the other hand, despite a reduction from over 1600 sequences described in the original study [11] down to just 600 still contain information about the role of Sierra Leone’s epidemic in maintaining transmission across the region through both endemic proliferation of lineages and their spread to neighbouring countries.

Unlike maximum likelihood phylogenies where branch lengths are directly proportional to the expected number of substitutions branch lengths in temporal phylogenies are usually smoothed out by the fact that a range of dates are compatible with a given number of mutations on a branch. Thus even large polytomies can be resolved into a branching structure derived from the tree prior albeit without much support for any given configuration. So despite the maximum likelihood of GP exhibiting a lot of polytomies (Fig. 1) the corresponding temporal phylogeny (left) does not, though it is more star-shaped than its genome equivalent with long external branches as well as numerous nodes with less than 0.10 posterior support (marked by grey crosses). Though the trees were too large to estimate robust tree distance statistics such as subtree prune and regraft (SPR) distances, Robinson-Foulds distances are not, and are smaller between the GP and genome maximum likelihood trees (188) than between Bayesian timetrees (1068). There are also noticeable differences in total tree length and whereas it is entirely expected that the maximum likelihood tree of GP should be larger (0.08076 substitutions/site) than the genome substitution phylogeny (0.06782 subs/site) due to the former’s faster rate of evolution the tree length of time trees differ 2-fold - 80.299 years for genome versus 173.018 years for GP. There is also a noticeable degree of branch clustering by country in the GP temporal phylogeny possibly caused by proximity of locations within country which in the absence of genetic information cannot be resolved to the same degree as with genomic data.

In contrast to the maximum likelihood phylogeny of GP on the right (Fig. 1) its corresponding temporal phylogeny on the left exhibits a reconstruction of the West African epidemic largely consistent with what has been established previously [11]. This is likely to be caused by the combined effects of two sources of information. First, additional information is added by specifying the collection dates for sequences which might exclude certain topologies from being considered during MCMC on account of the relatively small effective population size of Ebola virus in West Africa. Second, the generalised linear model approach to inferring migration is information-rich as it provides over 3000 possible parameter values (pairwise migration rates between locations) per predictor matrix and thus if a few branches are strongly selecting for a “correct” predictor matrix to be included in the migration model that predictor matrix can then be used to determine the likely locations of branches for which less information is available. However, a simpler continuous time Markov chain model where each individual pairwise migration rate is inferred individually in a maximum likelihood framework exhibits broadly similar patterns too (Additional file 2).

#### Loss of temporal information

**Fig. 2:**
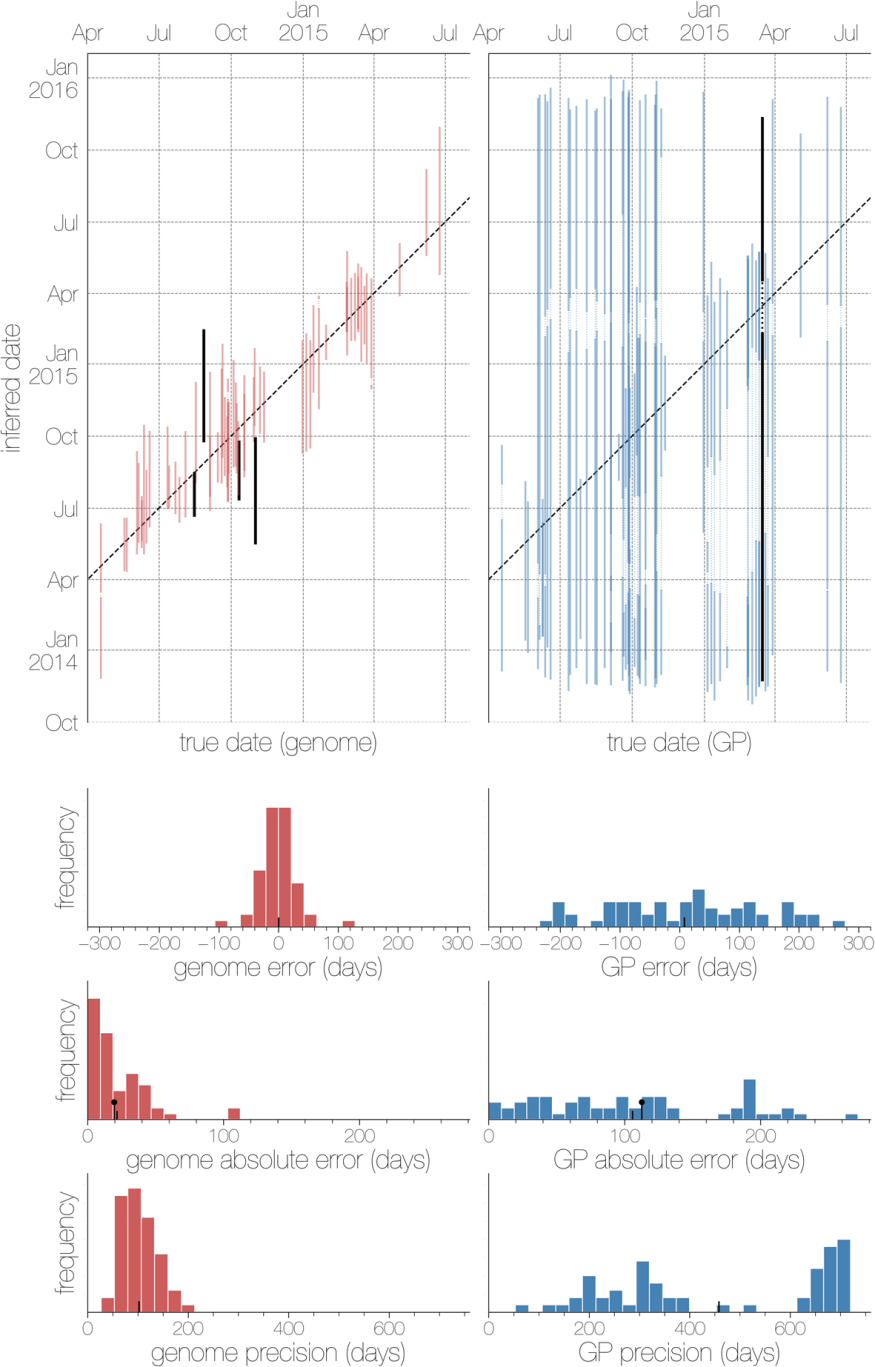
Masked tip date inference from genomes (left) and GP sequences (right). Inferred collection dates in the masked set based on genomes (red, left) and GP sequences (blue, right). Each vertical line corresponds to the 95% highest posterior density for the inferred tip date (y-axis) coloured red (genome) or blue (GP) if it falls within the true collection date (x-axis) and black otherwise. Dashed diagonal line indicates the 1-to-1 line. A histogram of signed residual errors between mean posterior date estimate and true date for each masked tip is shown in the second row with the black hatch indicating the mean. A histogram of *absolute* residual errors (accuracy) between mean posterior date estimate and true date for each masked tip is shown in the third row with the black hatch indicating the mean and the higher black hatch topped with a circle corresponding to a theoretical expectation based on Eq 2. Fourth row shows the histogram of confidence interval widths for date estimates (precision)

Inferring masked tip dates from 10% of the sequences (Fig. 2) is an intuitive way to show both the inherent noisiness of molecular clock estimates as reflected in the width of 95% highest posterior density intervals for inferred dates and the differences in temporal resolution between GP and genome alignments. True collection dates for genomes are mostly (56 out of 60, corresponding to a coverage probability of 0.93) within the 95% highest posterior density (HPD) of estimated dates and the mean absolute error is ≈22 days across all masked tips. In contrast the 95% HPDs for inferred dates in the GP dataset capture more of the true dates (58 out of 60, coverage probability ≈0.96) at the cost of markedly reduced precision with mean absolute error going up to ≈106 days or ∼3.5 months. Despite having lower coverage probability more precise date estimates are derived from complete genomes with an average 95% HPD width of ≈102 days compared to ≈458 days for GP. Another way of thinking about where the loss of information occurs is to consider root-to-tip against tip date regressions shown in Additional file 3 where waiting times for mutations are too long to estimate the slope of the regression reliably as every new mutation is seen across sequences collected over a longer interval of time. Observed errors (Fig. 2 but also Additional file 4 for maximum likelihood equivalent) for both datasets are very close to theoretical expectations calculated using Eq. 2: 22 (observed) versus 20 (expected) days for Ebola virus genomes and 106 (observed) versus 113 (expected) days for GP. Also note that for many tips in the GP data set independent Markov chains in some cases converged on, and in other cases sampled from, different distributions for masked tip dates (i.e. local maxima) resulting in multi-peaked posterior samples after combining independent analyses.

#### Migration model is strongly informed by tip dates and locations

**Fig. 3:**
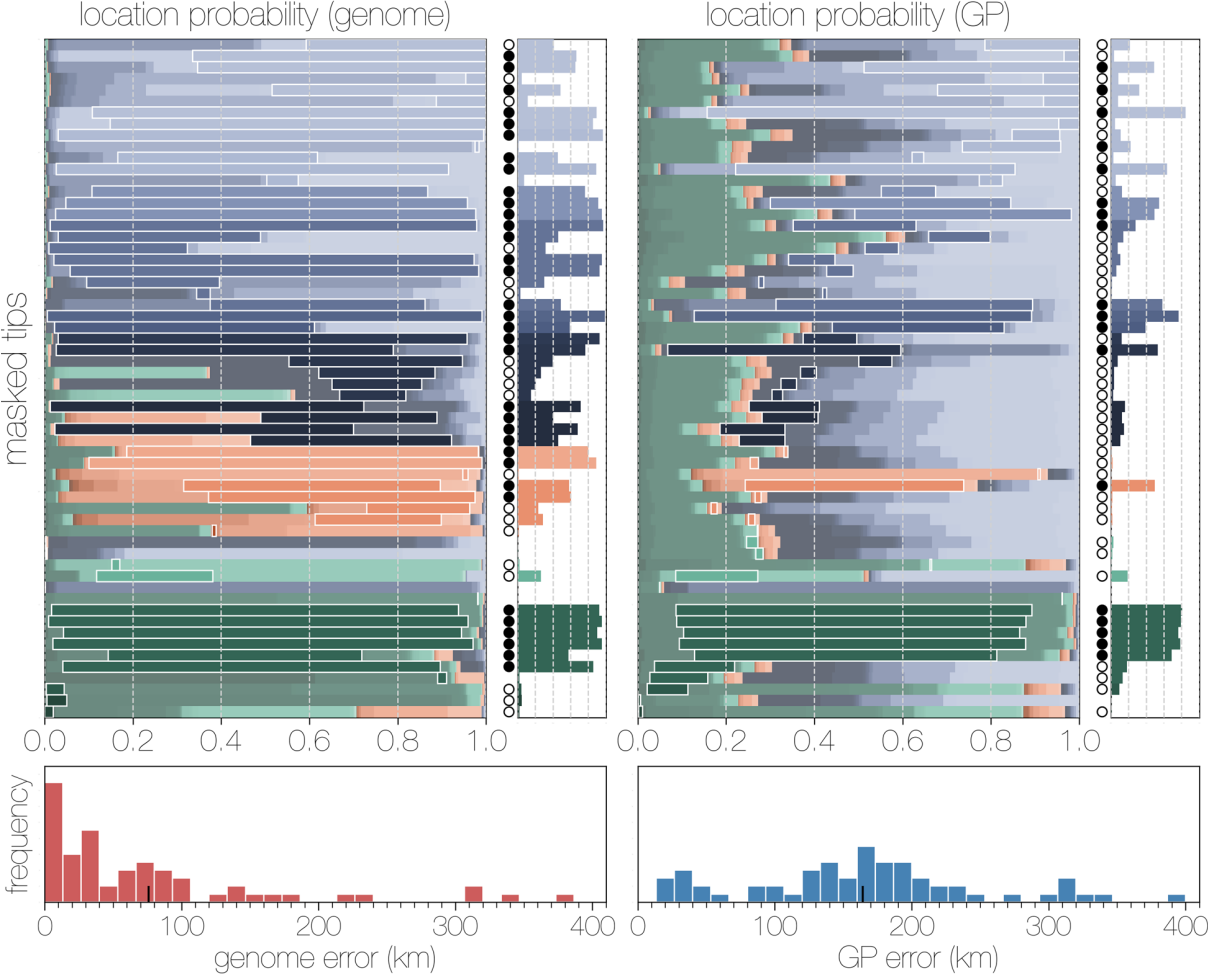
Masked tip location inference from genomes (left) and GP sequences (right). Horizontal bars indicate the posterior distribution of masked tip locations, coloured by country (Sierra Leone in blue, Liberia in red, Guinea in green) and location (lighter colours indicate administrative divisions lying towards west of the country). The correct location of each tip is outlined in white with the smaller plot to the right showing only the posterior probability of the correct location. Bars marked with an open circle indicate cases where the correct location is within the 95% credible set and solid circles indicate cases where the location with the most probability mass is also the correct location. Posterior distribution of probability-weighted distances between population centroids of inferred and correct locations is shown at the bottom with mean indicated by a tick mark

Differences between genomic and GP datasets are clear and dramatic when looking at both phylogenies (Fig. 1) and masked date inference (Fig. 2) but less pronounced when trying to infer the location of a masked tip (Fig. 3). Although locations are correctly inferred more often and with greater support in genomic sequences compared to just the GP gene there are numerous tips whose locations are not correctly inferred even from genome sequences (Fig. 3 and Additional file 5 for maximum likelihood equivalent). This might reflect the nature of these parameters of interest since phylogenies and date inference ultimately draw information from mutation accumulation via relatively straightforward models of sequence evolution with limited parameter space. In contrast, migration processes are far more complicated and nuanced without a *de facto* standard for modelling though continuous time Markov chain (CTMC) approaches are widely used with most advanced methods relying on generalised linear models without excessive over-parameterisation. Despite the lack of strong contrast in power to infer masked locations between genomes and GP sequences cross entropies indicate better performance with complete genomes (6054.631 nats) than with GP (9905.726 nats).

Similarly, locations are inferred correctly more often with complete genomes than with GP sequences where the maximum probability location (i.e. the model’s best guess) matches the truth. Specifically, using complete genomes results in 0.540 probability of guessing correctly compared to 0.286 probability for GP (for a calibration of both models see Additional file 6). The model makes these guesses with more certainty too where the mean probability of the true location is 0.482 with genomes and 0.219 with GP and mean probability of best guess (i.e. maximum probability) is 0.680 and 0.396, respectively. We also calculated the great circle distances between the population centroids of true and each predicted location weighed by probability which should ideally be 0.0 (0 km distance multiplied by probability of 1.0). The mean of these distances across masked tips are 75.886 kilometres for genomes compared to 164.309 km for GP sequences.

In addition to assessing how well tip locations can be inferred from genetic information we also looked at how well historical patterns were reconstructed from sequence data. To accomplish this we looked at the posterior distribution of ancestral locations of lineages that gave rise to four sequences in the data. The four lineages were chosen for their well-characterised histories in the broader epidemic as well as complexity of migration. Note that when we describe the movements of these lineages they are referred to by the strains that would eventually descend from them but it should in no way be interpreted as a single virus or patient moving around the region throughout the epidemic. Instead, all four strains share a number of common ancestors (the tree that would describe their relationships can be represented as (((‘14859_EMLK’, ‘MK3462’),‘EM_004422’),‘PL5294’);) whose locations can be inferred from the geographic distribution of their relatives and descendants.

Of the four representative sequenced viruses chosen three (‘14859_EMLK’, ‘MK3462’, ‘EM_004422’) are descended from the viral lineage that jumped across the border from Guéckédou prefecture in Guinea into Kailahun district in Sierra Leone. The common ancestor of two of those (‘14859_EMLK’ and ‘MK3462’) continued onto Kenema district in Sierra Leone from Kailahun district (also Sierra Leone). Unlike the lineage/transmission chain that eventually gave rise to strain ‘14859_EMLK’ which continued onto Conakry prefecture in Guinea and back-spilled into Sierra Leone’s Kambia district (where the descendent strain ‘14859_EMLK’ was collected [19]) right across the border later in the epidemic, the lineage/transmission chain that was ancestral to ‘MK3462’ stayed in Sierra Leone for the remainder of the epidemic and found itself moved westwards towards Freetown (Western Urban and Western Rural districts) until finally jumping to Bombali district where its descendent strain was collected from a patient [19]. Unlike all the other three lineages the transmission chain/lineage (“lineage A” [20]) that would leave strain ‘PL5294’ as a descendent is thought to have been largely restricted to the environs of Conakry prefecture in Guinea. An older ancestor of strain ‘PL5294’ had migrated from Guéckédou prefecture into Conakry prefecture on the other side of the country relatively early in the epidemic where its descendents circulated for a large portion of the West African epidemic though unlike a lot of its relatives the direct ancestor that would eventually give rise to strain ‘PL5294’ spilled over into Kambia district of Sierra Leone [19]. Finally, the transmission chain/lineage that would eventually give rise to strain ‘EM_004422’ had a tumultous history in the region. Strains ‘EM_004422’, ‘14859_EMLK’ and ‘MK3462’ all shared the same common ancestor in Kailahun district of Sierra Leone but the progenitor of strain ‘EM_004422’, unlike its relatives, made a jump to Liberia (Lofa and Montserrado counties) from where *its* descendants spilled back into Macenta prefecture in Guinea. This transmission chain would later leave descendants (of which ‘EM_004422’ is representative) that jumped to neighbouring Kissidougou prefecture (also Guinea) and where strain ‘EM_004422’ was collected [20].

**Fig. 4:**
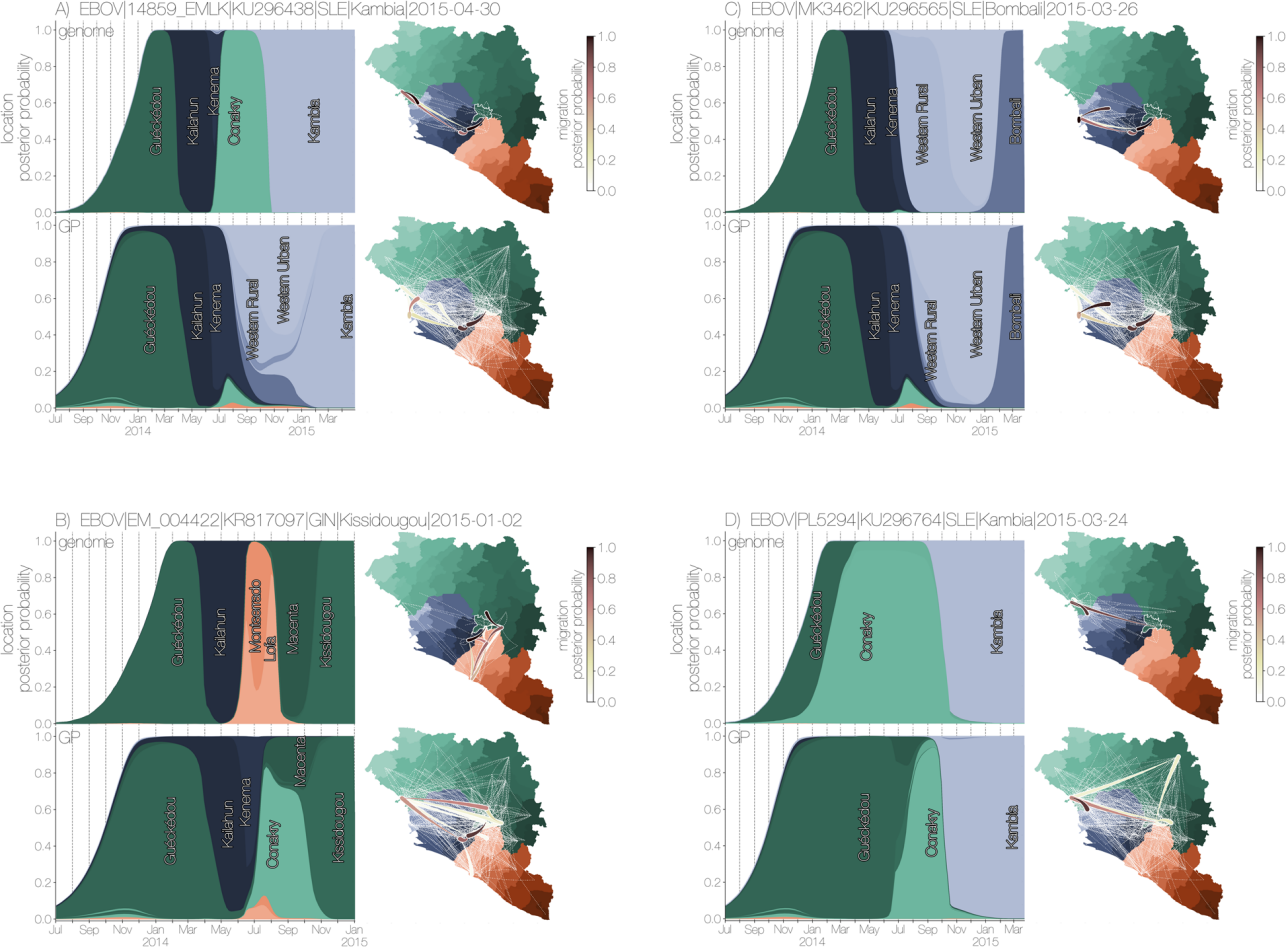
Posterior traces of ancestral locations and posterior migrations for four Ebola virus lineages from genomes (top) and GP sequences (bottom). The inferred ancestral branch location is logged at time points along the path from selected tips to the root of the tree across the posterior distribution of trees. The smoothed trajectories are an indication of where and when a lineage that gave rise to a particular tip is inferred to have existed. Maps on the right show migration events that are inferred to have taken place coloured by their posterior probability with migrations with <0.05 posterior support are shown as dotted white lines. All lineages share a common ancestor in Guéckédou prefecture of Guinea (white outline in the map) where the original zoonotic transmission event occurred near the Guinean border with Sierra Leone and Liberia. Some lineages are also descended from an early spillover event into Sierra Leone

The histories of the lineages that gave rise to these four tips are for the most part reconstructed from both GP sequences and genomes consistently (Fig. 4) likely as a result of additional information brought in by specifying tip dates and their collection locations. Genomic data tend to concentrate the probability mass towards a single location at any given time in contrast to GP sequences where several locations can be considered with non-negligible probabilities at numerous time points (Fig. 4 and Additional file 7) and where timing of ancestral migration events is considerably more diffuse or even substantially off (i.e. Fig. 4a and d). What is even more apparent is that without the additional information available when using complete genomes and without aiding the sampling with strongly informative priors MCMC explores a wider variety of low-probability migration paths as indicated by maps on the right of each plot in Fig. 4. In the case of the ancestral lineage of ‘EM_004422’, for example, a series of migrations through distant Conakry (western Guinea) are reconstructed with relatively high confidence from GP sequences compared to shorter distance migrations that run through neighbouring Liberia reconstructed from genomes.

**Fig. 5:**
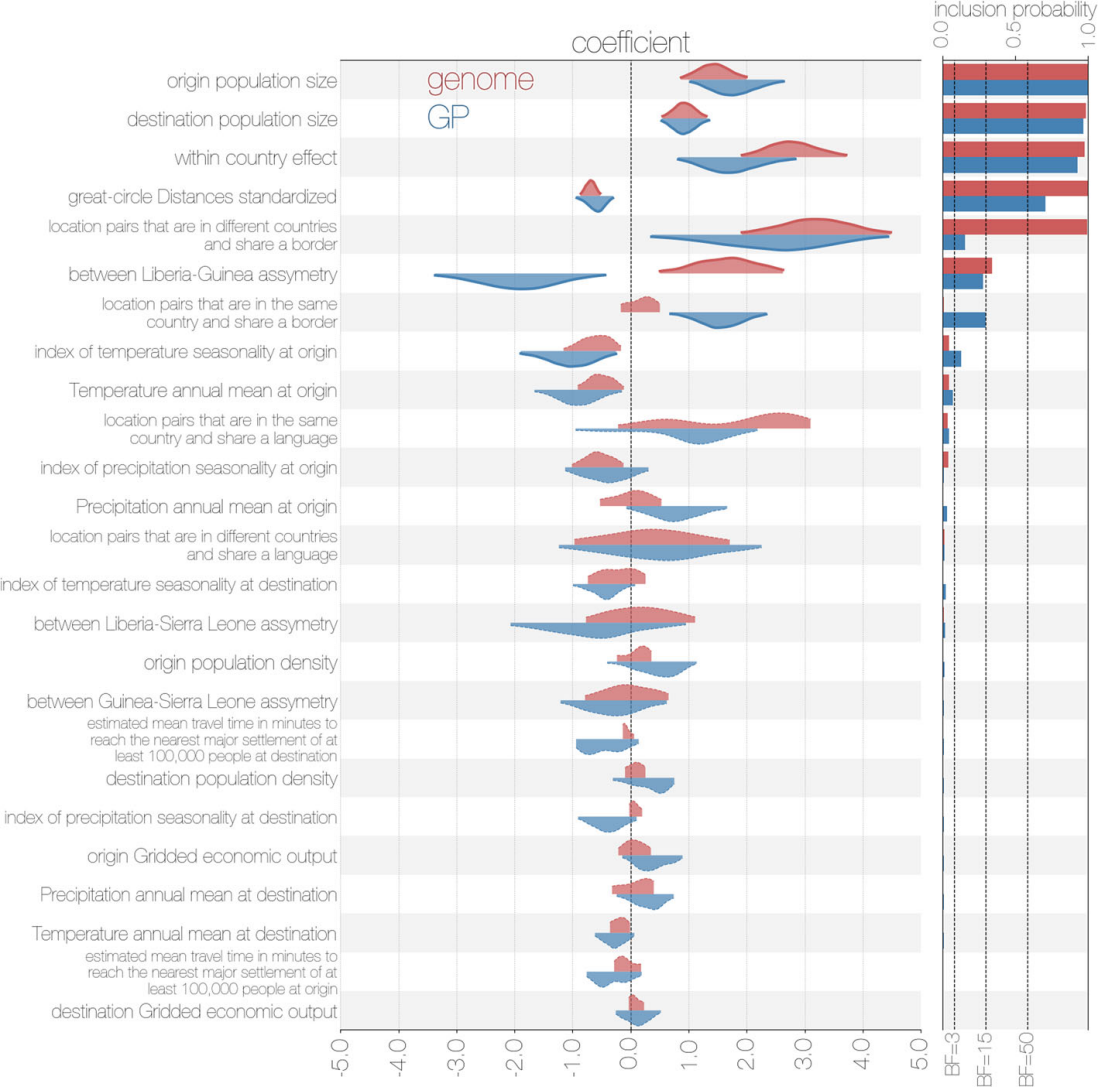
Correlates of migration identified from genomes (red) and GP sequences (blue). Effect size and direction of correlation between predictor matrices and migrations are shown as half violin plots where the top kernel density estimates (in red) are derived from genomes and bottom kernel density estimates (in blue) are derived from GP sequences conditioned on the predictor matrix being included in the model. Kernel density estimates of coefficients where predictors have <3 Bayes factor support are outlined in dashed lines. Posterior inclusion probabilities are shown on the right (red for genomes, blue for GP sequences) with appropriate Bayes factor cutoffs indicated by dashed lines

Despite markedly reduced information content for both total number of sequences (>1600 to 600) and additional loss of information in GP (≈90% fewer sites) sequences the same core correlates of migration are recovered for both datasets in the generalised linear model (Fig. 5) compared to previous findings using all available sequence data. These are: population sizes at origin and destination locations, within country migration effect, and great circle distances which are identified as strong predictors of migration with high (>50 Bayes factor, BF) albeit not categorical support (Fig. 5). Four other migration predictors for the GP dataset have support >5 BF and <15 BF which are international and national border sharing, Liberia-Guinea asymmetry, and index of temperature seasonality at origin. Of these Liberia-Guinea asymmetry and international border sharing are also found to be good predictors of migration in genomic data though confusingly Liberia-Guinea asymmetry has the opposite correlation sign with GP sequence data. Apart from this deviation predictors for both genome and GP gene datasets mostly have the same sign and very similar effect sizes. As mentioned previously (Figs. 1 and 4) this suggests substantial amounts of information being derived from collection dates and locations of tips rather than genetic information. The reduction in total numbers of sequences as well as reduced phylogenetic information in the GP dataset appears to enable the migration model to explore combinations of predictors that would otherwise be confidently excluded with complete genomes and thus a larger number of predictor matrices is included in the migration model with low probabilities. The differences between genomic and GP data though seemingly small (e.g. Fig. 3) is more pronounced when looking at total entropy of inclusion probabilities: 1.285 nats for genome data, and 2.688 for GP sequences.

#### Temporal resolution

As discussed in the introduction the mean waiting time for a mutation is 1/*RL* (Eq. 2) and depends on the rate at which mutations arise and are sampled by sequencing (evolutionary rate, *R*) and number of sites under observation (alignment length, *L*). Since 1/*RL* defines a linear relationship between rate *R* and length *L* mean waiting times for mutation can be reduced by an increase in either *R* or *L*. In order to double temporal resolution one can either double the evolutionary rate *R* or double the alignment length *L*. The former is generally outside the researchers’ control though genomic regions evolving at a faster rate exist in many pathogens. How much faster smaller regions evolve will depend on forces of population genetics such as ability to recombine with respect to the rest of the genome (strength of Hill-Robertson effect [21]) as well as positive selection or functional constraints. It is thus unlikely that significantly higher rates will offset the reduction in resolution caused by focusing on a very small genomic region. Extending the region that is sequenced, on the other hand, is often trivial outside of resource-limited areas and can dramatically improve temporal resolution.

**Fig. 6:**
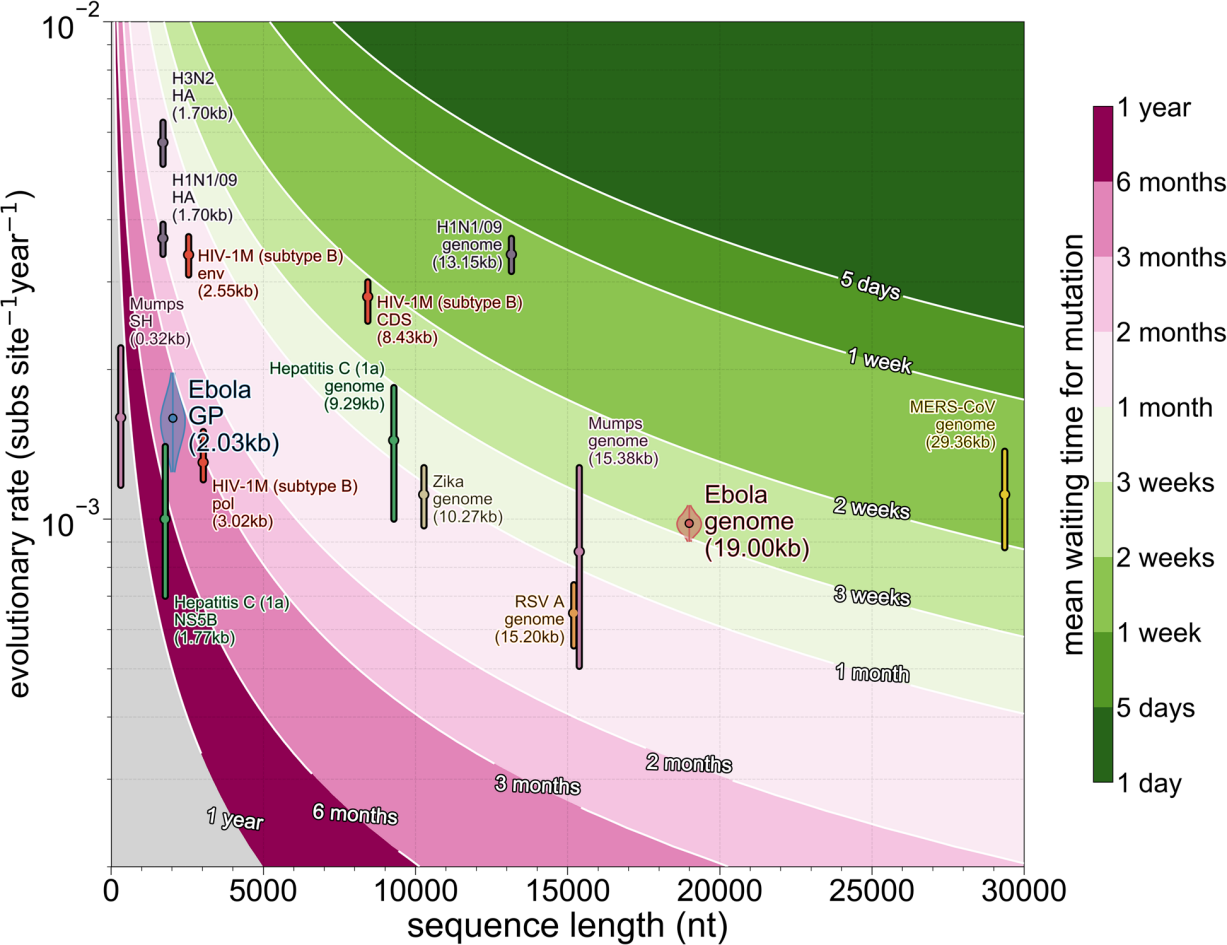
Mean waiting times for a mutation as a function of alignment length and evolutionary rate. Contours correspond to mean waiting times under a given combination of alignment length and evolutionary rate. Genomes and barcode genes for a variety of viruses are shown with reported evolutionary rate confidence intervals (vertical lines) and including analyses of Ebola virus genomes (red violin) and GP sequences (blue violin) reported here. Most genomes occupy parameter space implying temporal resolution of a mutation once every month or so. Subgenomic fragments on the other hand are expected to have mean mutation waiting times of more than a month

To help researchers intuit the impact of sequence length and evolutionary rate on temporal resolution we show the relationship between evolutionary rate and alignment length in determining mean waiting times until a mutation is observed in Fig. 6. In addition to theoretical expectations we also show where a variety of viral pathogens fall along the two axes - estimated evolutionary rates with uncertainty intervals on the y-axis and alignment length on the x-axis. Subgenomic alignments shown in Fig. 6 include the small hydrophobic (SH) gene of mumps virus [22] and glycoprotein (GP) sequences of Ebola virus analysed in this study as well as sequences of two human influenza A viruses: genome of subtype H1N1/09 [23], and haemagglutinin sequences of subtypes H1N1/09 [4] and H3N2 [24], sequences of two commonly studied blood-borne pathogens: *pol*, *env*, and coding regions of HIV-1M [25] and nonstructural protein 5B (NS5B) region of hepatitis C virus [26]. With respect to temporal resolution influenza A virus haemagglutinin gene (HA) is expected to acquire a mutation every one to two months on average compared to around three to six months for Ebola virus GP and over a year for mumps virus SH. Though all of these sequences are from (-)ssRNA viruses SH and GP genes are part of a single non-recombining RNA genome [27] whereas HA genes of influenza A viruses are encoded on their own segment which can be unlinked from their genomic background via reassortment. Because Ebola and mumps virus genomes do not recombine their polymerases may have been selected for higher fidelity due to Hill-Robertson effect.

Complete genomes, on the other hand, occupy parameter space that implies that a new mutation occurs on average every month or every few weeks. This is achieved through having more sites rather than substantial differences in evolutionary rates, which differ only marginally with respect to subgenomic fragments. Despite this no virus is expected to acquire mutations faster than about once per week on average and the two genomes with highest predicted temporal resolution - Middle East respiratory syndrome coronavirus (MERS-CoV) and H1N1/09 - are difficult to analyse due to recombination and reassortment, respectively, though advances are being made in modelling reticulate evolution [28]. The inverse relationship between observed evolutionary rate and sequence length is similar but not the same as the relationship between virus genome sizes and mutation rates where high mutation rates and large genome sizes lead to substantial deleterious mutation load [29]. This upper limit on mutation waiting times set by optimal evolutionary rates is what we refer to as the temporal horizon - population processes with inverse of rate (i.e. waiting time) less than the rate at which a pathogen acquires mutations will not be captured with high fidelity by currently existing methods. The exact relationship between mutation waiting times and rates of processes will of course be complicated by the presence of co-circulating lineages, site-wise rate heterogeneity, and choice of model for population processes of interest.

## Discussion

***Theoretical considerations***

For studies focused on temporal dynamics of pathogens over shorter periods of time the waiting time for a mutation should ideally be smaller than the inverse of the rate at which a process of interest occurs. Serial interval is often of most interest usually and has been addressed previously [30, 31] but migration or cross-species transmission rates could also exceed the critical temporal resolution threshold if sequences are assigned to compartments that are too small, i.e. the granularity of the analysis is increased by using a larger state space in the model. It is likely that this resolution limit will be improved greatly in the future by including additional information, either some aspects of a known transmission tree or more likely pathogen variation at the within-individual level where variant sharing between two or more individuals is evidence of their linkage in a transmission cluster. Much like evolutionary rates these methods might encounter biological limits outside of researchers’ control, however.

In addition to emphasising the need to sequence complete pathogen genomes we also hope that our study imparts the interpretation of pathogen evolutionary rates as primarily a parameter indicating temporal resolution of sequence data rather than a parameter of particular biological relevance. There have been previous incidents were a misunderstanding of the relationship between evolutionary rates and alignment length has been used to argue that low within-outbreak divergence in Ebola virus GP during the outbreak in Kikwit (Democratic Republic of Kongo) in 1995 was evidence of “genetic stability” [32]. What is far more likely to have taken place, however, is the phenomenon we show with our GP data (Fig. 1 and Additional file 1) where even after more than two years of the West African epidemic the GP gene is too short to accumulate appreciable numbers of mutations. Higher reported evolutionary rates early in the West African epidemic [33] have also been misreported as having biological meaning though not by the original study [34, 35] and arose through intense sequencing of a single transmission chain where mildly deleterious viral variants might not have been purged by purifying selection. We hope that our study clarifies that evolutionary rates are primarily a parameter of statistical resolution rather than of evolutionary forces and on their own are not sufficient to correctly interpret molecular clock data. Ideally, in the future sequence length and elapsed time will be included next to evolutionary rate estimates in order to transparently communicate statistical power available for analysis.

There is an additional Bayesian phylogenetic argument to be made in favour of using complete genomes. Molecular clock phylogenetics often relies on Markov chain Monte Carlo sampling to approximate the posterior distribution of phylogenetic trees [36]. Sequences which fall into polytomies in substitution phylogenies (i.e. well-defined common ancestry but no indication of exact branching order) are particularly problematic since plausible temporal phylogenies can be reconstructed in the absence of mutations. The branching order of such clades in time trees will be determined via the tree prior since no information about branching order can be recovered from the sequences themselves. There are over 34 million possible rooted trees for a set of 10 sequences but many of these might not be visited during MCMC if, for example, sequences are collected over time and effective population size (*N*_*e*_) is low. Nonetheless, MCMC is particularly inefficient at sampling tree topologies for identical sequences [37] since increasing the number of identical sequences leads to expansion of search space without adding additional information that could constrain the search. Until reliable methods are developed and standardised the current solution is to reduce the numbers of identical sequences going into temporal MCMC analyses.

***Practical considerations***

As well as temporal resolution concerns raised previously there are practical issues to consider when sequencing pathogens. Although many pathogens have established “barcode” genes or regions [38] some do not. This can easily lead to different groups sequencing different pathogen genes by chance or choice as has happened with Ebola virus previously where GP [39], a short fragment of the polymerase [40], or nucleoprotein [41] were sequenced which is not necessarily a problem when sufficient complete genomes are available to bridge information between disparate regions and appropriate methods of analysis are used [42]. Sequencing complete pathogen genomes in addition to providing the best possible resolution temporally in terms of mutation content (Fig. 6) also ends up aiding in standardising data between studies in the sense that a sequenced genome is a complete unit of data and there is nothing more to be done for sequence data except gathering better metadata.

It is also worth considering that the lifetime of sequence data extend beyond publication. Most scientific studies are designed with specific questions in mind that guide how data are collected and analysed to improve the researchers’ ability to detect differences. This makes combining data across studies with different goals (and correspondingly different data and approaches to analysing them) challenging. Sequence data on the other hand only become difficult to combine when sequences are too diverged to reliably align or are too numerous to infer phylogenies in reasonable time. Since divergence levels are generally low within outbreaks (with exceptions [43]) sequence data are often trivial to combine. More than that, including sequence data from previous studies can reciprocally contextualise both older and newer sequences (e.g. [44]). What remains problematic is determining and standardising additional data pertaining to the sequences themselves (“metadata”) in a way that makes sequence data easy to use by other groups. Whilst date and location of collection are widely reported and often of most interest non-standard encodings of both are seen in public databases.

***Stating the obvious***

As phylodynamic approaches are increasingly being applied to non-viral organisms it is important to set a good example of best data generation and analysis strategies. Sequencing complete bacterial genomes should lead to temporal resolution values comparable to those of viruses. For example *Enterococcus faecium*, evolving at a reported genomic evolutionary rate of 9.35×10^−6^ substitutions per site per year [45] and with a genome length of around 3.2Mb, is expected to experience at least one mutation in its genome every $\frac {1}{9.35 \times 10^{-6} \times 3.2 \times 10^{6}} \times 365$ days =12.2 days. These values are 4.41 years for *Mycobacterium tuberculosis* (evolutionary rate 5.67×10^−8^ subs/site/year [45] and a genome 4Mbp long) and 53.6 days for *Staphylococcus aureus* (evolutionary rate 2.43×10^−6^ subs/site/year [45] and a genome 2.8Mbp long) though care should be taken with evolutionary rate estimates as these are often reported per variable sites instead of genomic sites. Recombination is also a common though not universal concern when it comes to bacterial phylodynamics.

We have shown that a relatively simple model of sequence differentiation (Eq. 2) exhibits good correspondence with empirical results (Fig. 2) and can be used as a back-of-the-envelope calculation to gauge the power of a phylodynamic analysis. The relationship defined by Eq. 2 also describes a serious drawback of using partial genomes, namely that maintaining the same temporal resolution with decreasing fractions of the total sites available in a genome requires the remaining sites to evolve at increasingly unrealistic rates. This relationship is reciprocal such that for a 90% reduction in alignment length a 10-fold increase in evolutionary rate is required to achieve the same temporal resolution when compared to a complete genome. It is not at all surprising then that reducing the number of alignment columns by nearly 90% from ≈19,000 nucleotides that comprise the entire Ebola virus genome down to around 2,000 nt of the GP gene results in severe loss of information even if this shorter region evolves at a faster rate. Here we have quantified this loss of information via several methods: raw phylogenetic resolution (Fig. 1), molecular clock signal (Fig. 2), and aspects of migration model (Figs. 3, 4, and 5), which are summarised in Additional file 8.

In most cases biological aspects of the data such as precise branching order and molecular clock resolution suffer from severe loss in temporal resolution (Fig. 2) whereas modelling of non-biological aspects of the data, i.e. migration, tend to be more robust (Figs. 3 and 5). This is very likely to be caused by temporal and geographic rather than genetic features of the sequence data [46]. A clustering of sequences from a particular location collected over a short period of time is likely to be a genuine outbreak cluster within a wider epidemic and in the absence of genetic information phylogeographic models tend to group sequences by location. This might explain why in many cases when comparing analysis results between genome and GP datasets statistical power in migration model remains disproportionately high despite retaining only 10% of available sites and mutations and results between the entire >1600 genome data set [11] are very similar to the reduced data set analysed here. On a similar note case numbers alone have been used to recover a gravity-like model for the spread of Ebola virus in West Africa [47] previously, further arguing that the clustering of cases in time and space contains sufficient information about the movement of Ebola virus in West Africa. The overall conclusion from our study as well as others [17] is that sequencing short genomic regions instead of whole genomes is an ill-advised practice for investigating infectious disease outbreaks in any appreciable detail across relatively short timescales.

## Methods

***Sequence data***

A publicly available dataset of 1610 Ebola virus genomes sequenced by various groups [19, 20, 33, 48–55] and systematised in [11] was filtered to remove sequences where over 1% of the genome sequence was ambiguous or the precise location down to administrative division was not available leaving 943 genomes. A set of 600 viral genomes were randomly sampled from the filtered dataset of 943 high quality genomes. Of the 600 genomes that were chosen for analysis 10% (60 genomes) were chosen for masking where for all subsequent analyses both the date and location were considered as unknown and inferred as latent variables. Date inference was constrained via a uniform prior bounded by 2013 December 01 and 2015 December 01 corresponding roughly to the presumed beginning of the epidemic in late 2013 and its end in autumn of 2015. Another dataset was generated by extracting the glycoprotein GP coding sequence (with padding inserted into the polymerase slippage site to bring it in-frame) from the complete genomes dataset resulting in an alignment 2031 nucleotides long.

***Bayesian analyses***

Both GP and genome datasets were analysed in BEAST v1.8.10 [56] under the generalised linear model (GLM) described previously [3, 10, 11] to infer the migration model. Sites in both GP and genome alignments were partitioned into codon positions 1, 2, and 3, with the genome analysis also including a partition comprised of non-coding intergenic regions. Each partition was assigned an independent HKY+ *Γ*_4_ [57, 58] substitution model. A relaxed molecular clock [59] with an uninformative CTMC reference prior on the mean [60] of the log-normal distribution was used as the clock model. A flexible skygrid tree prior [61] was used to infer estimates of effective population size across 100 evenly spaced points in time starting 1.5 years prior to the collection of the most recent sequence to the date of the most recent sequence.

Both analyses (genome and GP) were set to run for 500 million states sampling every 50,000 states and run three (genome) or seven (GP) times independently. Due to technical issues with computational resources many analyses were not able to run to completion and so for full genomes only 136.5, 86.2, and 143.8 million states were sampled though after combining independent chains effective sample size (ESS) values are nearly the recommended 200. With the worst ESS values being prior (78) and precision (87) of GLM random effects, tree height (123), and prior (192), though largely as a result of bad mixing rather than convergence to different posteriors. Inference of masked tip dates often had poor ESSs as well mostly because of bad mixing and one example where all three chains independently sampled from two posterior distributions.

Similarly for GP only two MCMC analyses ran their allotted 500 million states with others running to 259.9, 253.9, 255.8, 261.65, and 261.5 million states. Unlike complete genome MCMC analyses GP analyses exhibit relatively poor ESS values even after combining seven independent chains which is indicative of bad mixing in the absence of additional data contained in complete genome sequences and uninformative priors. Poor ESS values amongst re-inferred tip dates are even more prevalent when using GP data and are primarily caused by both convergence of independent chains onto different stationary distributions and individual chains sampling distinct distributions. Worst ESSs for other parameters were: likelihoods for the three codon positions (48, 31, and 48 for positions 1, 2 and 3, respectively), overall likelihood (60), joint/posterior (98), coefficient of variation (104), standard deviation of lognormal distribution from which branch rates are drawn (107), alpha parameter of gamma distribution used to model rate heterogeneity across second codon positions (155), tree prior (166), overall prior (177), and effective population size estimate at the earliest grid point (190).

Convergence, mixing and appropriate burn-in values were assessed with Tracer v.1.7 [62] where 50 million states from every analysis (genome and GP) was discarded as burnin with GP data additionally subsampled down to a quarter of the sampled states. Log files of analyses are available on GitHub at https://github.com/blab/genomic-horizon/blob/master/data/xml/logs.zip and traces for posterior, prior and their product (called posterior) probabilities are shown in Additional file 9.

Posterior distributions of inferred tip dates for the masked set were logged during MCMC and 95% highest posterior density intervals were computed using a custom Python script due to multi-peaked posterior distributions after combining independent analyses. Briefly, the script takes a kernel density estimate of posterior samples and computes the integral of the peaks intersected by a horizontal line that is lowered until the integral of the peaks intersected encompasses 0.95 of the area. Posterior distributions of trees were summarised as maximum clade credibility (MCC) trees using TreeAnnotator [56]. Inferred posterior probabilities of masked tip locations were recovered from MCC trees. Ancestral location probabilities were recovered via a script called samogitia.py with the ‘ancestry’ option (available at https://github.com/blab/genomic-horizon/blob/master/scripts/samogitia.py) across 200 equally spaced time points between mid-2013 and beginning of 2016. The script samogitia.py uses baltic (available at https://github.com/evogytis/baltic) to parse posterior MCMC trees generated by BEAST.

***Maximum likelihood analyses***

RAxML [63] was used to infer maximum likelihood phylogenies for genome and GP datasets under the same partitioning as described for Bayesian analyses: three codon position partitions for GP and genome with genomes having an additional partition for intergenic regions under independent GTR+CAT substitution models. Trees were rooted in TreeTime according to best r^2^ value for root-to-tip against collection date regression with the 2 year constraint used for masked tips described earlier. A temporal phylogeny with marginal reconstruction of most likely dates for masked tips was carried out in TreeTime [64] as well. Ancestral sequences at internal nodes of the clock-rooted RAxML topology were inferred using TreeTime under an HKY model [57] of evolution. Ancestral location states were inferred in TreeTime using a continuous time Markov chain model identical to the one used by [2] without the Bayesian stochastic search variable selection. We also repeated many of the analyses under a maximum likelihood model in TreeTime [64] like inference of masked tip dates (Additional file 4) and locations (Additional file 5).

***Error computation***

For Fig. 2 mean absolute errors were computed as

3 $$ \epsilon = \frac{1}{N} \left(\sum_{i=1}^{N} \left(| t_{i} - \frac{1}{M} \sum_{m=1}^{M} e_{i}|\right)\right)  $$

Where N is the number of masked tips, *t*_*i*_ is the true date of the *i*th masked tip, *e*_*i*_ is the estimated date of the *i*th masked tip, and M is the number of states sampled from the posterior distribution.

For Fig. 3 errors expressed in units of distance were calculated as

4 $$ \epsilon = \frac{1}{N} \left(\sum_{i=1}^{N} \left(\sum_{j=1}^{J} \Delta(t_{i},e_{j})\times p_{ij}\right)\right)  $$

Where N is the number of masked tips, J is the number of locations in the migration model, *Δ* is great circle distance in kilometres, *t*_*i*_ is the coordinate of the population centroid of the true location of the *i*th masked tip, *e*_*j*_ is the coordinate of the population centroid of *j*th location, and *p*_*ij*_ is the probability that the *i*th tip is in *j*th location.

Entropies for predictors shown in Fig. 5 and location probabilities in Additional file 7 were calculated as

5 $$ S = -\sum_{i} P_{i} {log}_{e}(P_{i})  $$

where *P*_*i*_ is the mean posterior inclusion probability of *i*th predictor matrix in the model for Figure 5 and probability of *i*th location for Additional file 7.

Cross entropies for Fig. 3 were calculated as

6 $$ H = -\sum_{i}^{N} {log}_{e}(q_{i})  $$

where N is the number of masked tips, *q*_*i*_ is the probability of the true location of the *i*th masked tip, which is assigned a probability of 0.0001 if the true location does not appear in the set of inferred possible locations (i.e. has probability 0.0) to avoid domain error.

## Supplementary information

**Additional file 1** Whole genome maximum likelihood tree coloured by mutations occurring in GP. Colours indicate the cumulative number of mutations from the root occurring in the GP gene. Much of the clade resolution is lost when only considering mutations occurring in the GP gene, particularly in the already highly polytomic Sierra Leonean part of the phylogeny in red.

**Additional file 2** Maximum likelihood phylogenies of complete Ebola virus genomes (left) and GP sequences (right) with maximum likelihood ancestral location reconstruction. Trees were inferred in RAxML [63] with ancestral state reconstruction performed in TreeTime [64]. Inferred phylogeographic patterns are for the most part consistent with Bayesian results presented in Fig. 1 with severe loss of statistical power when using GP instead of genome sequences.

**Additional file 3** Root to tip regression for maximum likelihood trees of genome (red) and GP (blue) sequences. Linear regression of sequence collection dates against distance from the root gives evolutionary rate estimates (slope of the regression) at 0.82×10^−3^ and 0.73×10^−3^ substitutions per site per year, respectively. Despite similar rates the correlation between collection dates and divergence from root is far better using genomes (*r*^2^=0.76) than GP sequences (*r*^2^=0.13).

**Additional file 4** Maximum likelihood inference of masked tip dates from genomes (red, left) and GP sequences (blue, right) using TreeTime. Vertical bars indicate the 95% confidence interval for marginal reconstruction of masked tip dates plotted against their true dates. Tip dates where the 95% confidence interval excludes the true value are shown in black.

**Additional file 5** Maximum likelihood inference of masked sequence location from genomes (left) and GP sequences (right) via a CTMC model implemented in TreeTime. Horizontal bars indicate the posterior distribution of masked tip locations coloured by country (Sierra Leone in blue, Liberia in red, Guinea in green) and location (lighter colours indicate administrative divisions lying towards west of the country). The correct location of each tip is outlined in white with the smaller plot to the right showing only the probability of the correct location. Bars marked with an open circle indicate cases where the correct location is within the 95% credible set and solid circles indicate cases where the location with the most probability is also the correct location. Genomes still perform better in terms of correct guess (0.432 probability that best guess location is true location for genomes versus 0.259 for GP), cross entropy (12012.800 nats for genome versus 24397.109 nats for GP) and mean probability-weighted great circle distance between true location population centroid and estimated location population centroid (87.568 km for genome versus 124.909 km for GP).

**Additional file 6** Calibration curve for phylogeographic model informed with genome (red) and GP (blue) sequences. Logistic regression of probability of the most likely location against whether it is correct or not for genome (red) and GP (blue) sequences with jitter introduced along the y axis to make points discernible. Overall performance of the phylogeographic model is comparable between genome and GP sequences as indicated by sigmoid curves matching the 1-to-1 dotted line.

**Additional file 7** Entropies of posterior ancestral location reconstruction from genomes (red) and GP sequences (blue) for four tips. Ancestral state reconstructions from genomes typically have lower entropies relative to reconstructions derived from GP sequences indicating better certainty in location assignment at any given time. Red and blue bars at the end of the plot indicate relative cumulative entropies of genome and GP sequence reconstructions, respectively.

**Additional file 8** Summary of statistics reported in this study. Each cell shows the difference between genome (red, bottom of cell) and GP (blue, top of cell) data for various statistics reported in this study. Descriptions for each statistic are given at the bottom of the cell near the x-axis.

**Additional file 9** MCMC traces of prior, likelihood and joint (referred to as posterior) probabilities. Post-burnin MCMC samples of prior, likelihood and joint probabilities for genome data (total of three chains, top) and GP data (total of seven chains, bottom) with kernel density estimates of each chain displayed on the right.

## Data Availability

Sequence data and all analytical code is publicly available at https://github.com/blab/genomic-horizon.

## Abbreviations

BEAST: Bayesian evolutionary analysis by sampling trees
BF: Bayes factor
CAT: Short for _categories
CTMC: Continuous time Markov chain
ESS: Effective sample size
GLM: Generalised linear model
GP: Glycoprotein
GTR: Generalised time reversible
H1N1: Haemagglutinin 1, neuraminidase 1
H3N2: Haemagglutinin 3, neuraminidase 2
HA: Haemagglutinin
HIV: Human immunodeficiency virus
HKY: Hasegawa-Kishino-Yano
HPD: Highest posterior density
km: Kilometres
Mb: Megabases
MCC: Maximum clade credibility
MCMC: Markov chain Monte Carlo
MERS-CoV: Middle East respiratory syndrome coronavirus
NS5B: Nonstructural protein 5B
nt: Nucleotides
SH: Small hydrophobic
ssRNA: Single-stranded ribonucleic acid
SPR: Subtree prune and regraft
subs: Substitutions
RAxML: Randomized axelerated maximum likelihood
RNA: Ribonucleic acid
