# Supplementary figures and images for: The ability of single genes vs full genomes to resolve time and space in outbreak analysis
Source: BMC Evol Biol. 2019 Dec 26;19:232. doi: 10.1186/s12862-019-1567-0 (PMC6933756; doi:10.1186/s12862-019-1567-0)

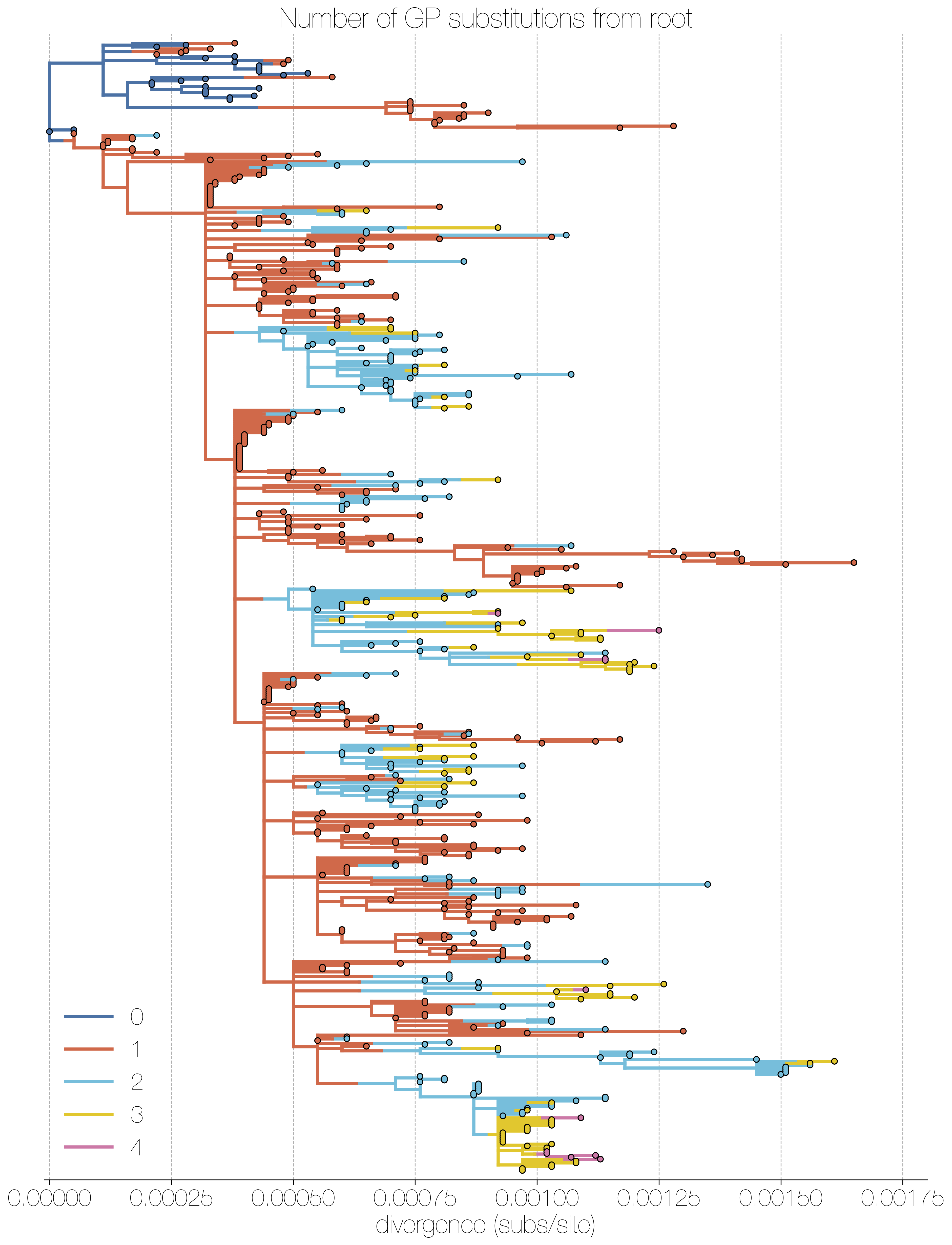

Supplement: Supplementary file 1 — Additional file 1 Whole genome maximum likelihood tree coloured by mutations occurring in GP. Colours indicate the cumulative number of mutations from the root occurring in the GP gene. Much of the clade resolution is lost when only considering mutations occurring in the GP gene, particularly in the already highly polytomic Sierra Leonean part of the phylogeny in red. [file 12862_2019_1567_MOESM1_ESM.png]

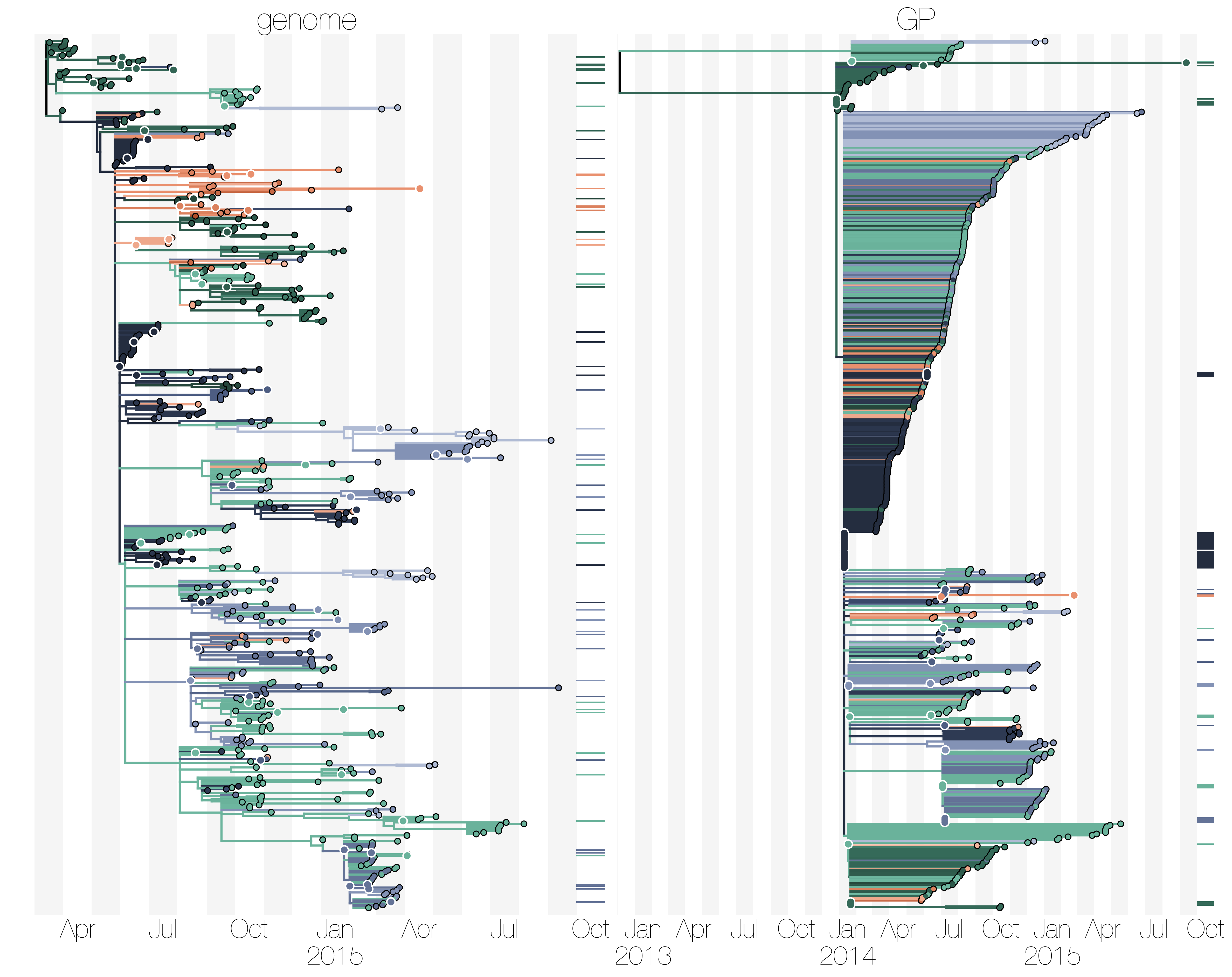

Supplement: Supplementary file 2 — Additional file 2 Maximum likelihood phylogenies of complete Ebola virus genomes (left) and GP sequences (right) with maximum likelihood ancestral location reconstruction. Trees were inferred in RAxML [63] with ancestral state reconstruction performed in TreeTime [64]. Inferred phylogeographic patterns are for the most part consistent with Bayesian results presented in Fig. 1 with severe loss of statistical power when using GP instead of genome sequences. [file 12862_2019_1567_MOESM2_ESM.png]

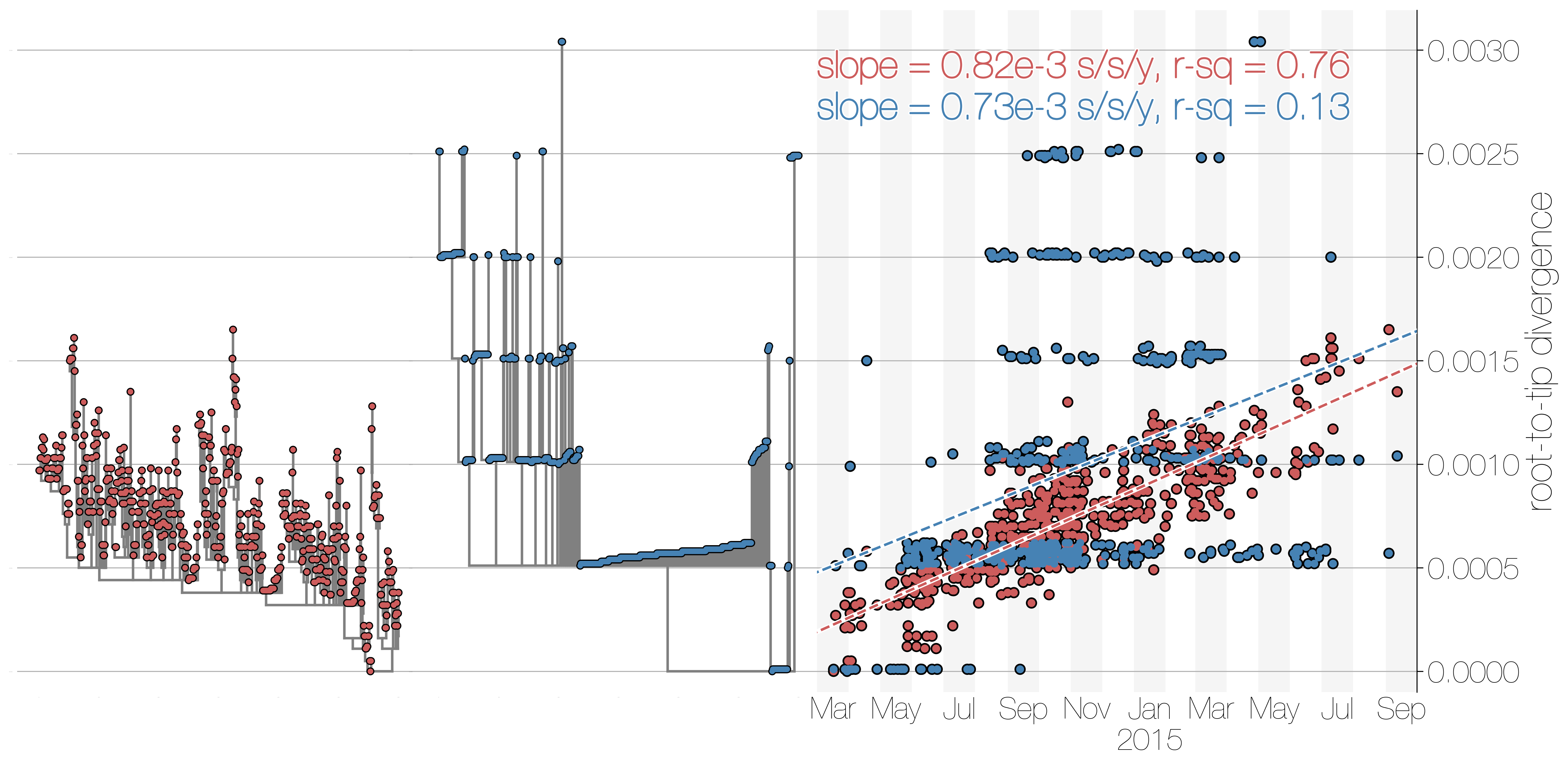

Supplement: Supplementary file 3 — Additional file 3 Root to tip regression for maximum likelihood trees of genome (red) and GP (blue) sequences. Linear regression of sequence collection dates against distance from the root gives evolutionary rate estimates (slope of the regression) at 0.82×10^−3^ and 0.73×10^−3^ substitutions per site per year, respectively. Despite similar rates the correlation between collection dates and divergence from root is far better using genomes (r2=0.76) than GP sequences (r2=0.13). [file 12862_2019_1567_MOESM3_ESM.png]

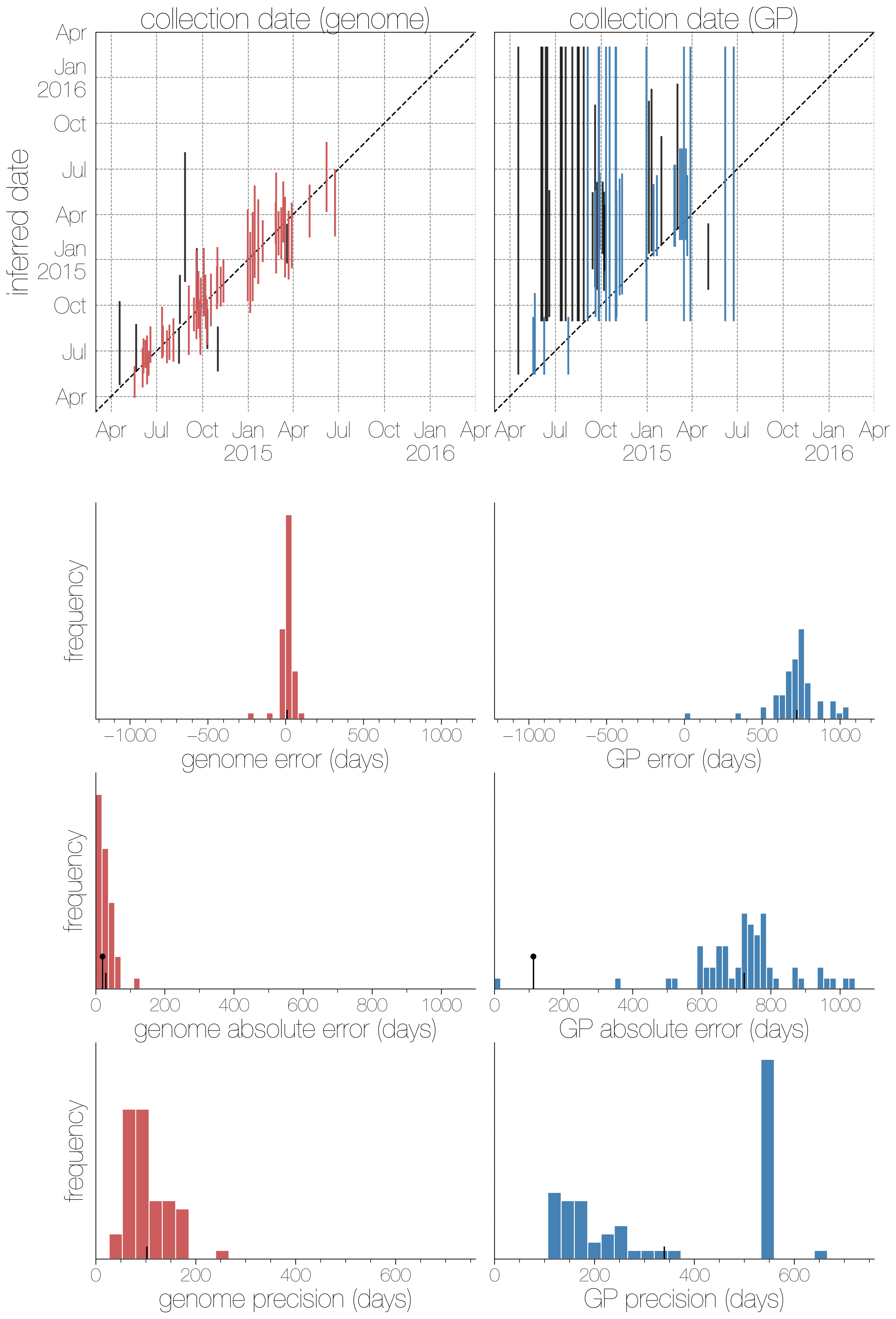

Supplement: Supplementary file 4 — Additional file 4 Maximum likelihood inference of masked tip dates from genomes (red, left) and GP sequences (blue, right) using TreeTime. Vertical bars indicate the 95% confidence interval for marginal reconstruction of masked tip dates plotted against their true dates. Tip dates where the 95% confidence interval excludes the true value are shown in black. [file 12862_2019_1567_MOESM4_ESM.png]

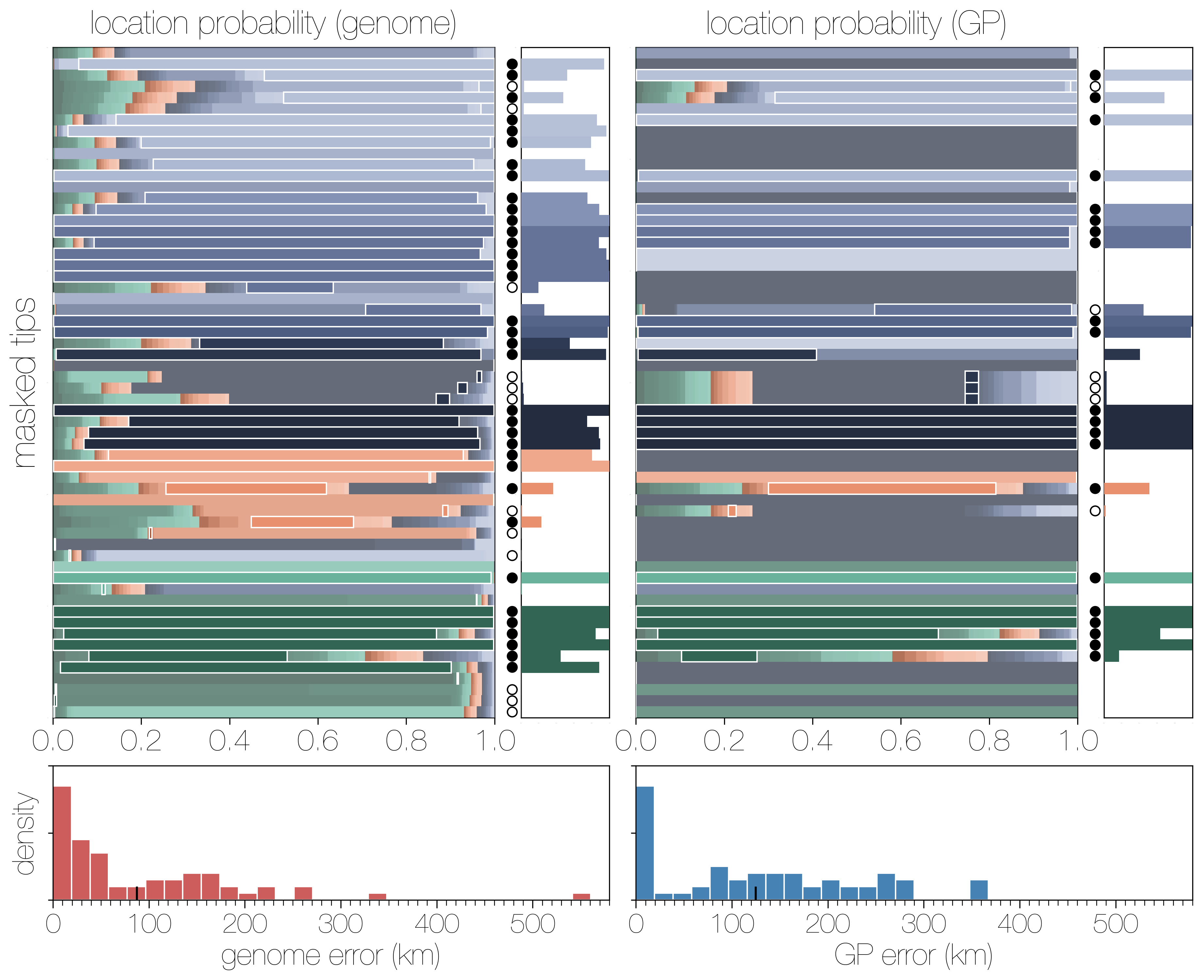

Supplement: Supplementary file 5 — Additional file 5 Maximum likelihood inference of masked sequence location from genomes (left) and GP sequences (right) via a CTMC model implemented in TreeTime. Horizontal bars indicate the posterior distribution of masked tip locations coloured by country (Sierra Leone in blue, Liberia in red, Guinea in green) and location (lighter colours indicate administrative divisions lying towards west of the country). The correct location of each tip is outlined in white with the smaller plot to the right showing only the probability of the correct location. Bars marked with an open circle indicate cases where the correct location is within the 95% credible set and solid circles indicate cases where the location with the most probability is also the correct location. Genomes still perform better in terms of correct guess (0.432 probability that best guess location is true location for genomes versus 0.259 for GP), cross entropy (12012.800 nats for genome versus 24397.109 nats for GP) and mean probability-weighted great circle distance between true location population centroid and estimated location population centroid (87.568 km for genome versus 124.909 km for GP). [file 12862_2019_1567_MOESM5_ESM.png]

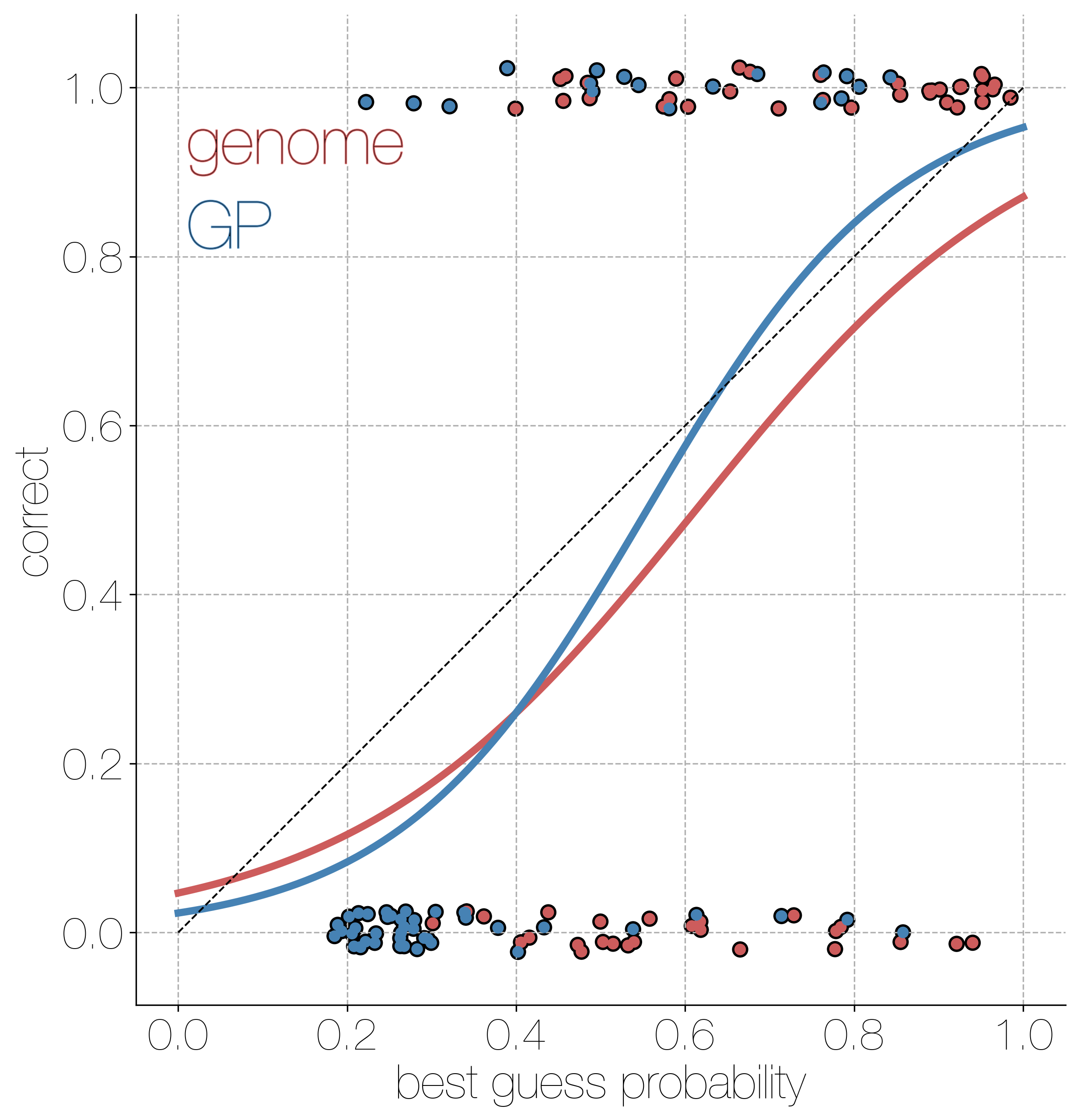

Supplement: Supplementary file 6 — Additional file 6 Calibration curve for phylogeographic model informed with genome (red) and GP (blue) sequences. Logistic regression of probability of the most likely location against whether it is correct or not for genome (red) and GP (blue) sequences with jitter introduced along the y axis to make points discernible. Overall performance of the phylogeographic model is comparable between genome and GP sequences as indicated by sigmoid curves matching the 1-to-1 dotted line. [file 12862_2019_1567_MOESM6_ESM.png]

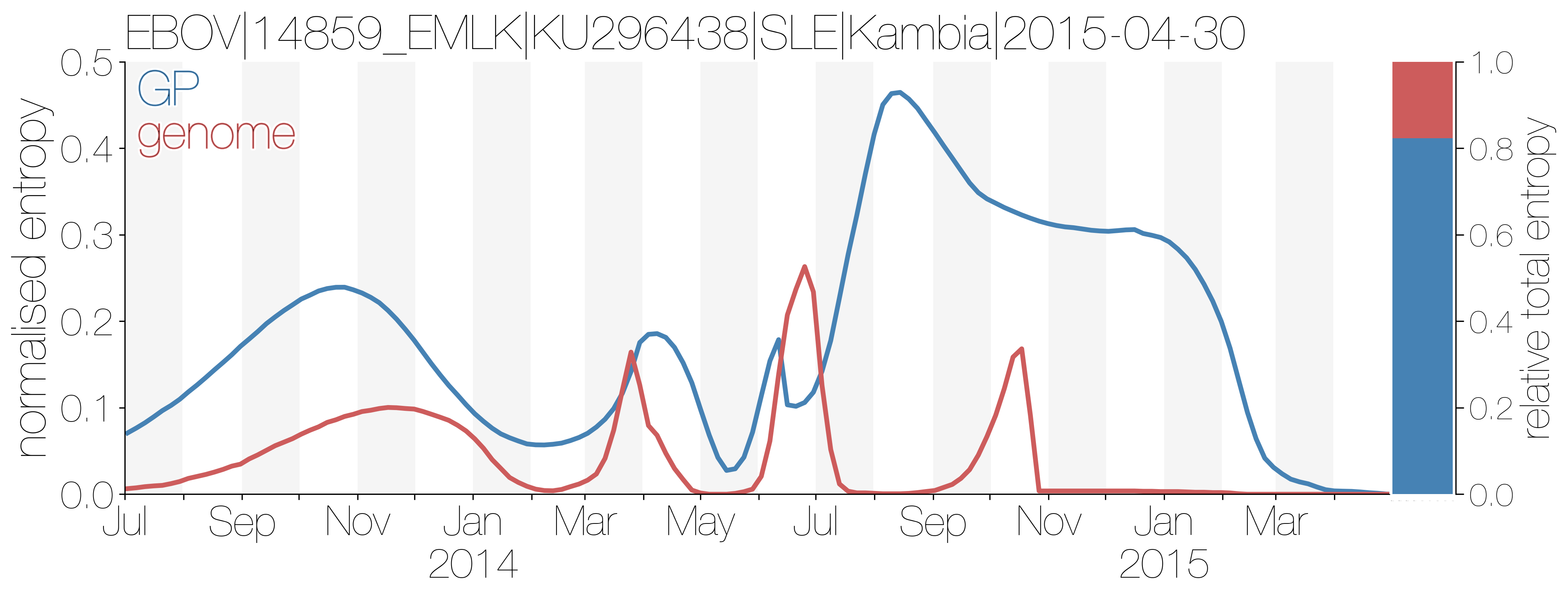

Supplement: Supplementary file 7 — Additional file 7 Entropies of posterior ancestral location reconstruction from genomes (red) and GP sequences (blue) for four tips. Ancestral state reconstructions from genomes typically have lower entropies relative to reconstructions derived from GP sequences indicating better certainty in location assignment at any given time. Red and blue bars at the end of the plot indicate relative cumulative entropies of genome and GP sequence reconstructions, respectively. [file 12862_2019_1567_MOESM7_ESM.zip › 12862_2019_1567_MOESM7_ESM/12862_2019_1567_MOESM7a_ESM.png]

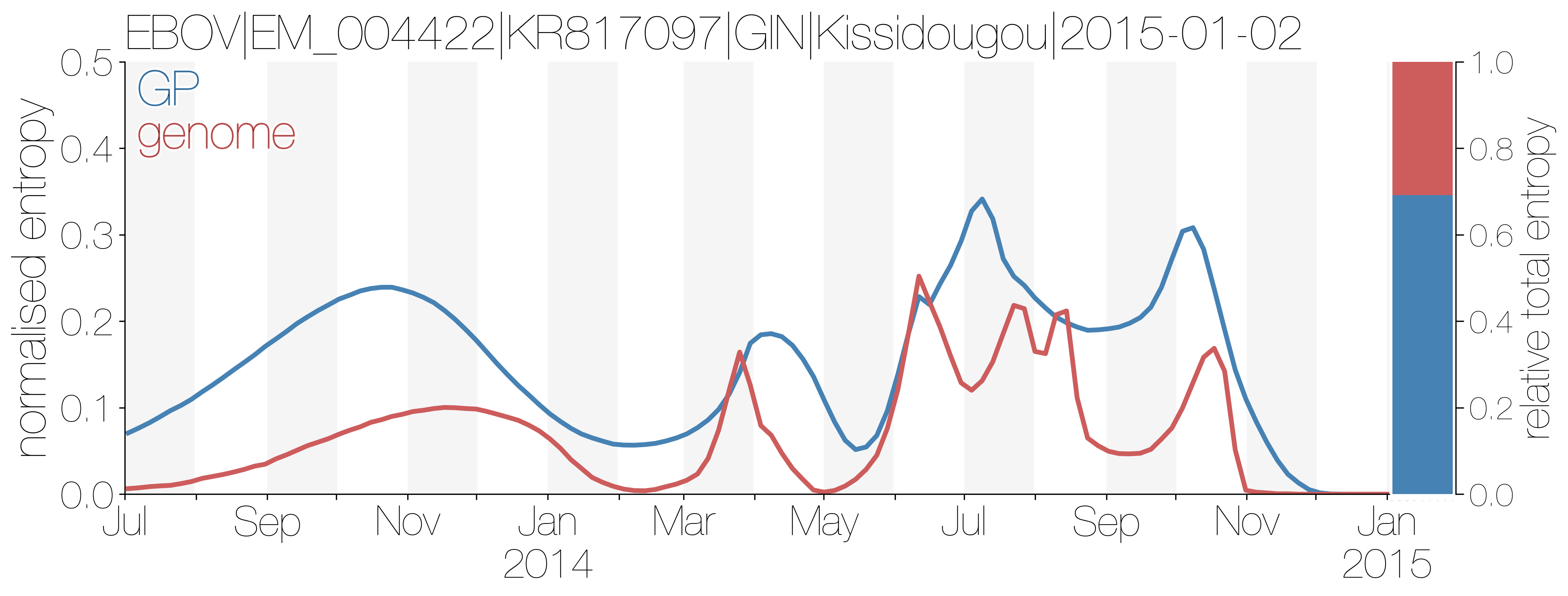

Supplement: Supplementary file 7 — Additional file 7 Entropies of posterior ancestral location reconstruction from genomes (red) and GP sequences (blue) for four tips. Ancestral state reconstructions from genomes typically have lower entropies relative to reconstructions derived from GP sequences indicating better certainty in location assignment at any given time. Red and blue bars at the end of the plot indicate relative cumulative entropies of genome and GP sequence reconstructions, respectively. [file 12862_2019_1567_MOESM7_ESM.zip › 12862_2019_1567_MOESM7_ESM/12862_2019_1567_MOESM7b_ESM.png]

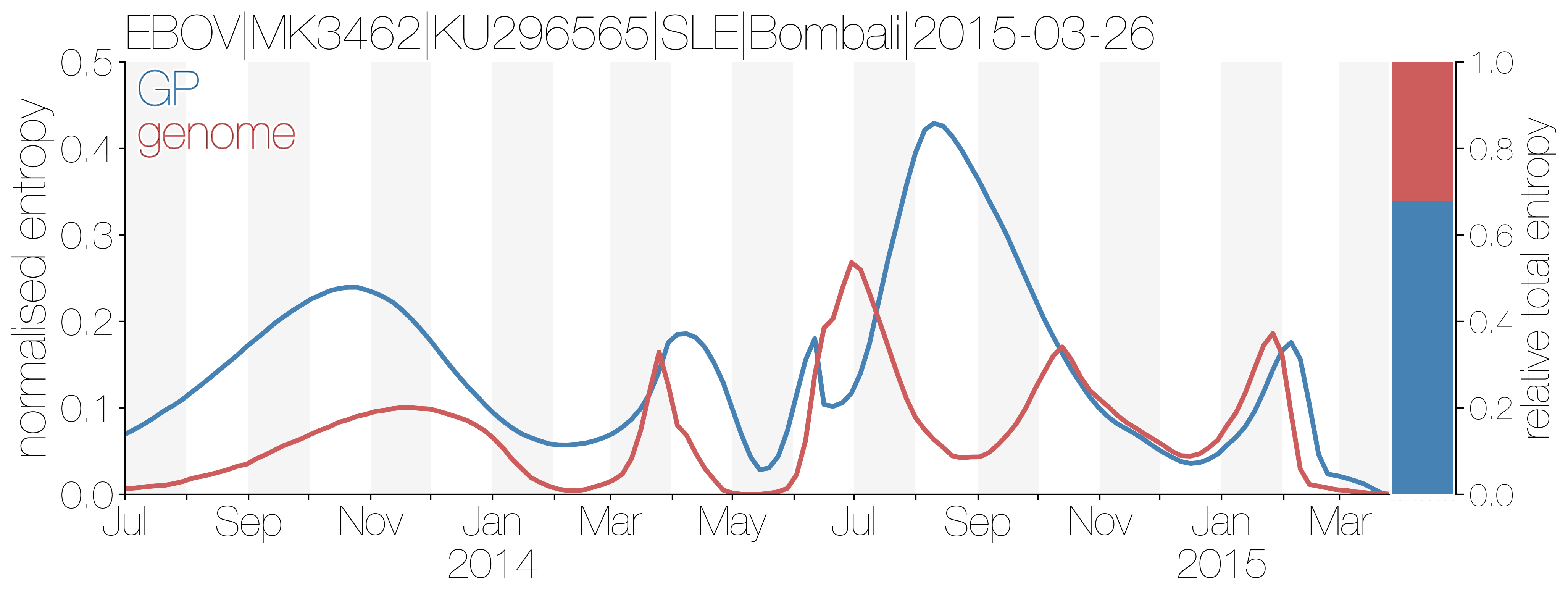

Supplement: Supplementary file 7 — Additional file 7 Entropies of posterior ancestral location reconstruction from genomes (red) and GP sequences (blue) for four tips. Ancestral state reconstructions from genomes typically have lower entropies relative to reconstructions derived from GP sequences indicating better certainty in location assignment at any given time. Red and blue bars at the end of the plot indicate relative cumulative entropies of genome and GP sequence reconstructions, respectively. [file 12862_2019_1567_MOESM7_ESM.zip › 12862_2019_1567_MOESM7_ESM/12862_2019_1567_MOESM7c_ESM.png]

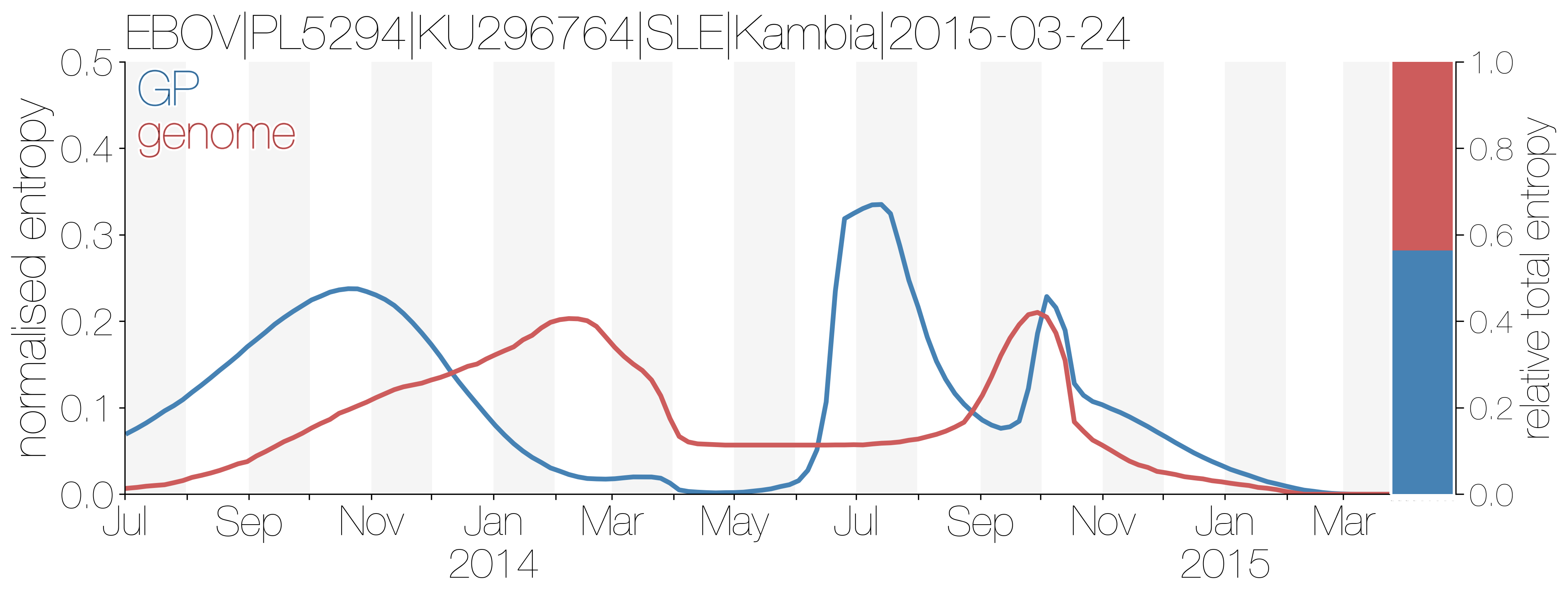

Supplement: Supplementary file 7 — Additional file 7 Entropies of posterior ancestral location reconstruction from genomes (red) and GP sequences (blue) for four tips. Ancestral state reconstructions from genomes typically have lower entropies relative to reconstructions derived from GP sequences indicating better certainty in location assignment at any given time. Red and blue bars at the end of the plot indicate relative cumulative entropies of genome and GP sequence reconstructions, respectively. [file 12862_2019_1567_MOESM7_ESM.zip › 12862_2019_1567_MOESM7_ESM/12862_2019_1567_MOESM7d_ESM.png]

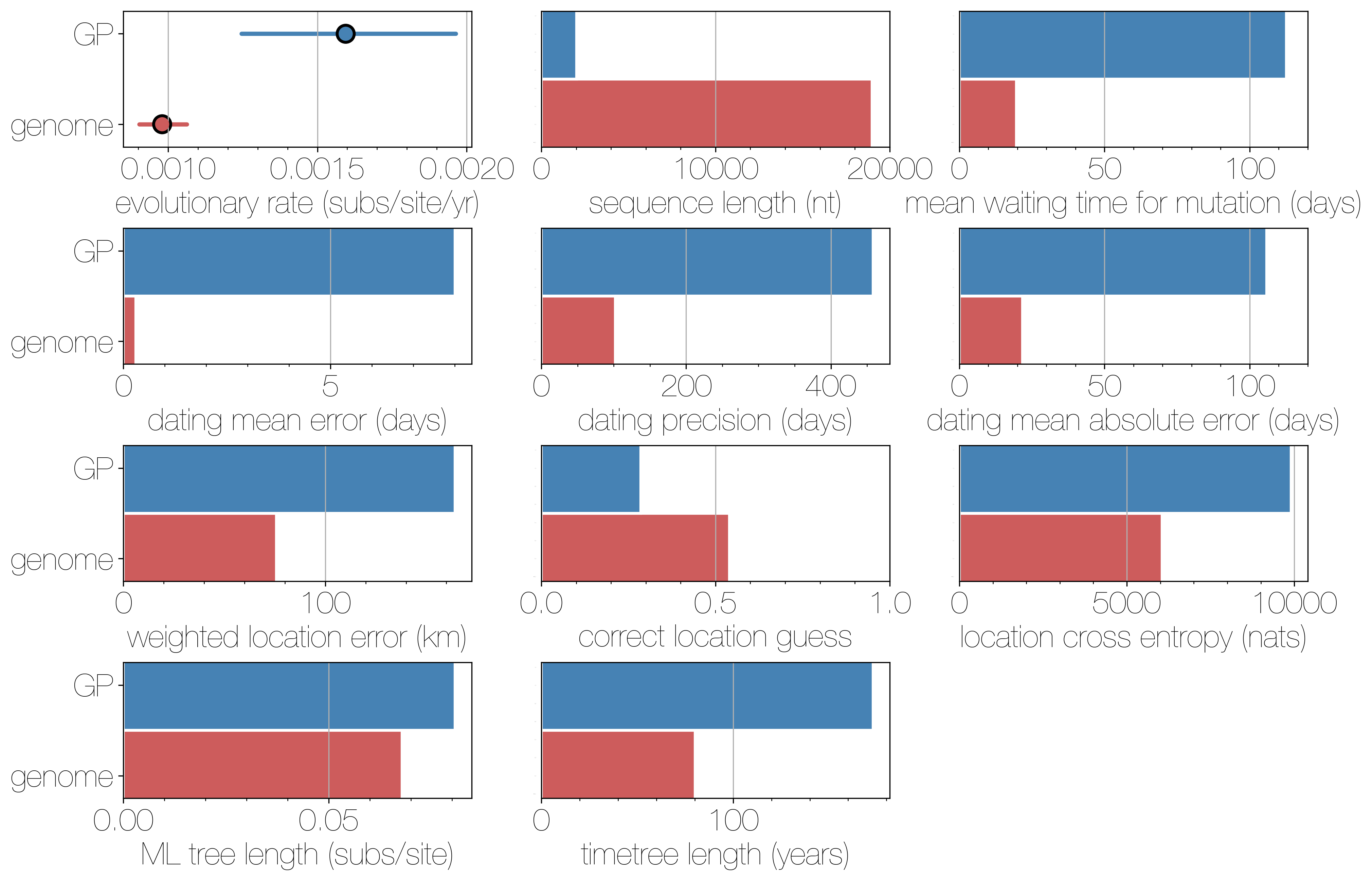

Supplement: Supplementary file 8 — Additional file 8 Summary of statistics reported in this study. Each cell shows the difference between genome (red, bottom of cell) and GP (blue, top of cell) data for various statistics reported in this study. Descriptions for each statistic are given at the bottom of the cell near the x-axis. [file 12862_2019_1567_MOESM8_ESM.png]

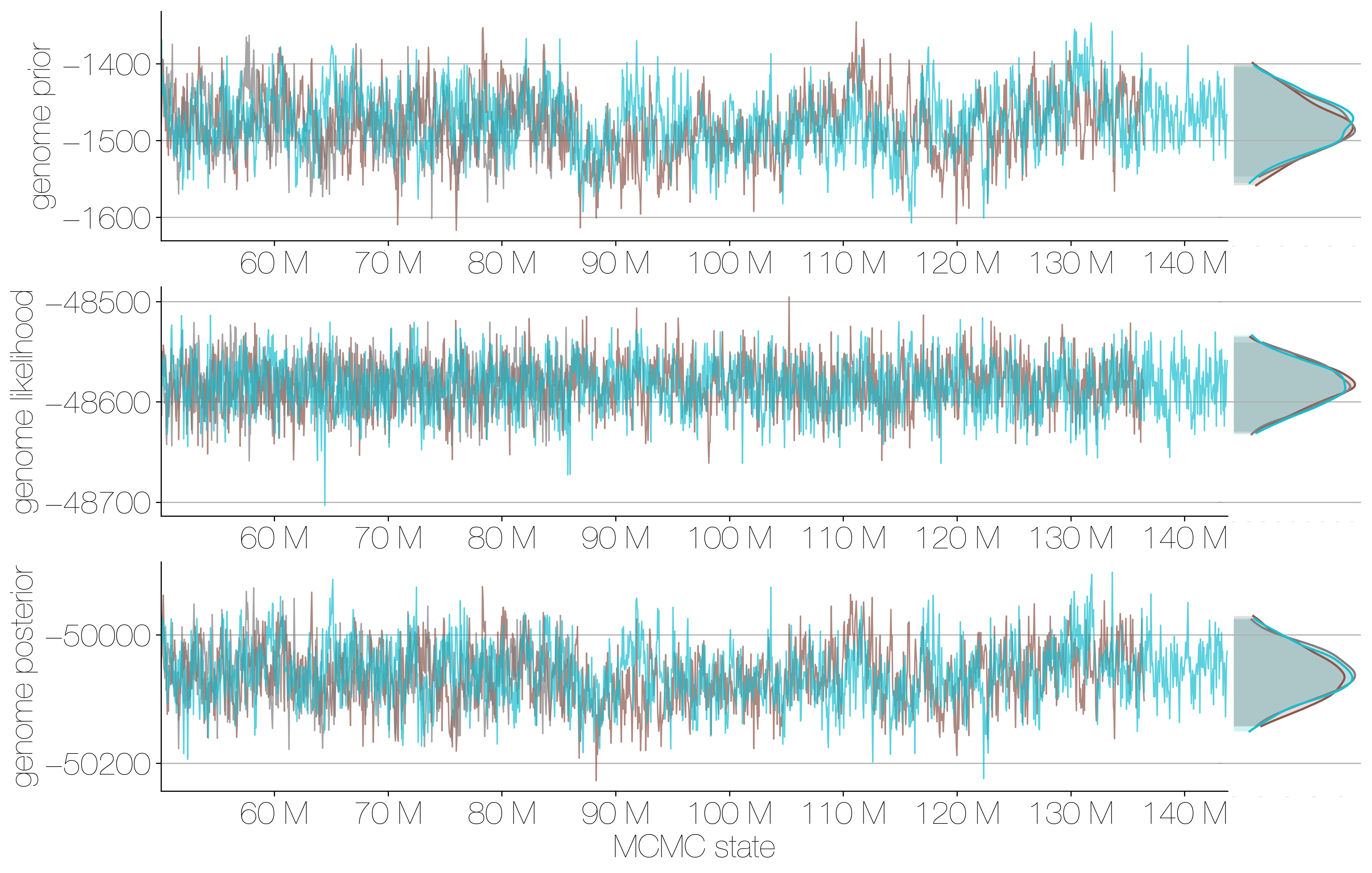

Supplement: Supplementary file 9 — Additional file 9 MCMC traces of prior, likelihood and joint (referred to as posterior) probabilities. Post-burnin MCMC samples of prior, likelihood and joint probabilities for genome data (total of three chains, top) and GP data (total of seven chains, bottom) with kernel density estimates of each chain displayed on the right. [file 12862_2019_1567_MOESM9_ESM.zip › 12862_2019_1567_MOESM9_ESM/12862_2019_1567_MOESM9a_ESM.png]

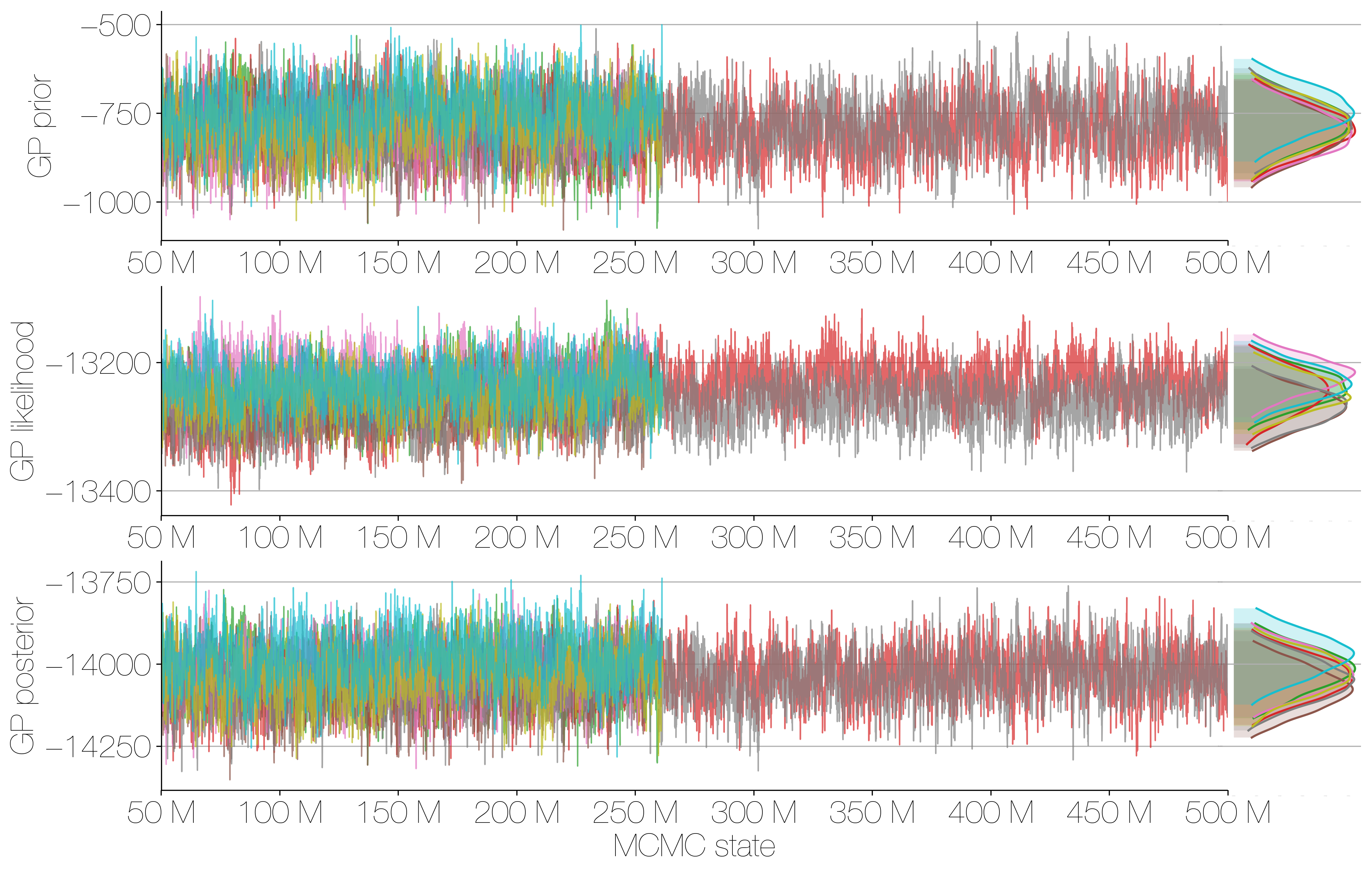

Supplement: Supplementary file 9 — Additional file 9 MCMC traces of prior, likelihood and joint (referred to as posterior) probabilities. Post-burnin MCMC samples of prior, likelihood and joint probabilities for genome data (total of three chains, top) and GP data (total of seven chains, bottom) with kernel density estimates of each chain displayed on the right. [file 12862_2019_1567_MOESM9_ESM.zip › 12862_2019_1567_MOESM9_ESM/12862_2019_1567_MOESM9b_ESM.png]
